# Effects of balneotherapy on stress, anxiety, and depression: results of a multicenter randomized controlled trial with six-month follow-up

**DOI:** 10.1186/s12906-026-05323-4

**Published:** 2026-03-17

**Authors:** Lolita Rapolienė, Aelita Bredelytė, Giedrė Taletavičienė, Arvydas Balčius, Jūratė Astravaitė, Jovita Jočienė, Virmantas Rinkevičius, Antanas Danys, Liucija Patinskienė, Nerijus Kemeklis, Beatričė Pargaliauskytė, Dovydas Rapolis, Justina Raulušonytė, Antonella Fioravanti

**Affiliations:** 1 Klaipėda University, Klaipėda, Lithuania; 2 Baltic Medics, Klaipėda, Lithuania; 3 Druskininkai Hospital, Druskininkai, Lithuania; 4 Health resort Draugystė, Druskininkai, Lithuania; 5 Medical SPA Eglės sanatorija, Druskininkai, Lithuania; 6 Gradiali Medical Spa & Wellness, Palanga, Lithuania; 7 Health resort Versmė, Birštonas, Lithuania; 8 Tulpės Sanatorium, Birštonas, Lithuania; 9 Recreation and health complex Atostogų parkas, Kretingos reg, Žibininkai, Lithuania; 10 Vilnius University, Vilnius, Lithuania; 11 Independent Researcher, Siena, Italy

**Keywords:** Balneotherapy, Complementary medicine, Stress reduction, Depression, Anxiety, Randomized controlled trial, Mental health promotion

## Abstract

**Background:**

Balneotherapy (BT), involving the use of natural resources such as mineral water, peloids, and climate exposure, is increasingly recognized as a complementary intervention for stress-related mental health conditions. This study aimed to evaluate the short- and long-term effects of a standardized BT complex on stress, anxiety, depression, and related outcomes.

**Methods:**

A multicenter, randomized, controlled, single-blinded (researchers) parallel-group trial was conducted in six Lithuanian medical spa centers. Adults aged 18–65 years with moderate to high stress levels (*N* = 243) were randomized into three intervention groups—ambulatory BT (11ABT), ambulatory BT plus nature therapy (11ABTNT), inpatient BT (11BTS)—and one control group (11 C). The treatment period was 11 days, with assessments at baseline, post-intervention, 3 months, and 6 months. Primary outcomes included perceived stress (PSS-10), salivary cortisol, anxiety (STAI-5), and depression (CESD-R). Secondary outcomes were fatigue (FAS), sleep quality (SQS), functional adaptation (WSAS), integrative well-being, cognitive performance, and treatment safety.

**Results:**

All intervention groups showed statistically significant improvements across primary and secondary outcomes compared to baseline (*p* < 0.001), with medium to very large effect sizes. The greatest short-term effects were observed in the inpatient group (11BTS), including reductions of 46% in stress intensity, 54% in depression, and 49% in sleep impairment. Improvements in fatigue, anxiety, and stress management were also notable. These effects persisted at 6 months, with sustained reductions of up to 31% in stress, 27% in anxiety, and 50% in stress management. Sleep quality improved by up to 67%, and integrative outcomes by 40%. No serious adverse events were reported, and treatment was well-tolerated across all sites.

**Conclusions:**

Balneotherapy using natural resources is a safe, effective, and sustainable complementary approach for improving mental health. The observed reductions in stress, anxiety, depression, and fatigue—alongside enhanced sleep and functioning—suggest that BT may be a valuable addition to integrative care strategies for stress-related conditions. Further large-scale trials are warranted to confirm these findings and support wider clinical implementation.

**Trial registration:**

ClinicalTrials.gov Identifier NCT06018649 (August 25th, 2023).

**Supplementary Information:**

The online version contains supplementary material available at 10.1186/s12906-026-05323-4.

## Introduction

Difficult life situations cause a state of worry or mental tension. The World Health Organization (WHO) identifies this mental state as stress. Stress is a natural human response that encourages us to deal with challenges and threats in our lives [1] but has a negative side, i.e., it influences our health and overall well-being [2], reduces productivity, and creates obstacles to achieving goals [3], being linked with seven of the ten leading causes of mortality worldwide [4].

It is estimated that 36.5% of the world’s population suffers from stress; half experience psychological distress, nearly a third suffer from depression, anxiety, sleep disorders, and low well-being, and 1.2% have attempted suicide [5]. Even before the COVID-19 era, the societal cost of mental health disorders exceeded 4% of GDP (over EUR 600 billion) across the 28 European Union countries [6]. In 2021, 4 in 10 adults worldwide said they experienced a lot of worry (42%) or stress (41%) [7]. Thus, stress and its associated mental health consequences represent a global public health concern, further exacerbated by the COVID-19 pandemic, which contributed to a 25% increase in anxiety and depression [8].

The brain is the central organ for stress - it determines how we interpret a situation and controls our behavioral and physiological response. Chronic stress disrupts neuroendocrine regulation, particularly through the hypothalamic-pituitary-adrenal (HPA) axis, which governs the release of cortisol, a key biomarker of the stress response [9]. Prolonged cortisol dysregulation is associated with impaired cognitive function, anxiety, depression, sleep disturbances, and metabolic disorders [10, 11]. Cortisol - sometimes referred to as a “window on the brain” - both reflects and influences brain structure and function, particularly in the prefrontal cortex, amygdala, and hippocampus, which are involved in mood, attention, and behavioral control [12, 13].

Although it remains unclear which biomarkers best reflect mental stress states, cortisol and adrenaline are widely studied [10, 11]. Salivary cortisol, in particular, is a validated, non-invasive biomarker of endocrinological stress response and can be used to detect early signs of psychological distress or to monitor the effectiveness of stress-reduction interventions [13].

Given the rising burden of stress-related mental disorders, there is growing demand for accessible and effective complementary interventions. Although pharmacological and psychotherapeutic approaches are standard, there are often unmet needs in care delivery [14]. Non-pharmacological methods such as art/music therapy, mindfulness, yoga, nature-based interventions, and physical activity have been shown to reduce stress and enhance psychological well-being [15].

Balneotherapy (BT) - the therapeutic use of mineral water, peloids (medicinal mud), and climate - is an established complementary therapy, especially in the management of low-grade inflammation and stress-related conditions such as rheumatic and metabolic disorders [16].

In addition to physical health benefits, increasing evidence suggests that balneotherapy has a positive impact on mental health outcomes, including anxiety and depression. It was found that BT was as effective as paroxetine in reducing symptoms of generalized anxiety disorder [17]. In another study, anxiolytic effects and modulation of oxidative stress in rodents following BT with low-dose radon exposure were reported [18]. A recent meta-analysis confirmed BT’s effectiveness in alleviating depression symptoms in fibromyalgia patients [19], while improvements in mood and sleep quality in healthy elderly individuals undergoing a short BT program were also demonstrated [20].

Its therapeutic effects likely result from a combination of mechanical, thermal, chemical, and neuroendocrine mechanisms [21]. Warm mineral baths may improve circulation and parasympathetic tone, while peloids are thought to possess anti-inflammatory and immunomodulatory properties. Immersion in warm mineral-rich water has also been shown to affect cortisol levels and support mental relaxation [21].

Recent research also supports BT’s positive influence on stress biomarkers and psychological well-being. A systematic review highlights the potential of balneotherapy to modulate cortisol levels, suggesting a role in regulating the hypothalamic-pituitary-adrenal (HPA) axis and improving stress resilience [22]. Similarly, it was demonstrated that spa therapy significantly alleviated mental stress, sleep disturbances, and general health complaints in sub-healthy individuals, further reinforcing BT’s utility for psychosomatic health [23].

Although BT is widely practiced, few studies have explored its long-term effects on stress and mental health using standardized psychological instruments and biological markers. Moreover, most previous studies are limited by small sample sizes, short intervention durations, and single-center designs. To our knowledge, large-scale multicenter trials evaluating both the physiological and psychological effects of BT - including salivary cortisol, perceived stress, depression, and anxiety - are lacking.

The beneficial effects of balneotherapy may be explained by its influence on both psychological and physiological mechanisms. Warm water and peloid treatments help activate the parasympathetic nervous system, reduce sympathetic activity, and promote relaxation. These changes can support stress recovery by modulating the hypothalamic-pituitary-adrenal (HPA) axis and reducing levels of cortisol, a key stress hormone. Therefore, cortisol measurement alongside psychological assessments provides valuable insight into the body’s response to balneotherapy.

The aim of this study was to evaluate the short- and long-term effects of a standardized balneotherapy complex on stress and related mental health indicators, including anxiety, depression, fatigue, sleep quality, and social functioning. A multicenter randomized controlled single-blinded trial was conducted to test the hypothesis that participants receiving BT would show significantly greater improvements in psychological and physiological stress outcomes - compared to a control group - and that these effects would be sustained over a six-month period.

Although previous studies have explored the short-term effects of balneotherapy on physical and psychological well-being, there is a lack of long-term, multicenter randomized controlled trials (RCTs) assessing its sustained impact on stress, anxiety, and depression. Furthermore, most existing studies do not evaluate BT as a person-focused, integrative intervention that may foster elements of mindfulness and self-regulation through immersion in natural environments and relaxation practices. This study aims to address these gaps by providing robust evidence from a multicenter RCT with six-month follow-up, combining subjective and objective stress-related outcomes.

We hypothesized that participants receiving balneotherapy would experience significantly greater reductions in stress, anxiety, depression, and fatigue, as well as improvements in sleep quality and psychological functioning, compared to the control group, with effects sustained over a six-month follow-up.

## Materials and methods

### Reporting standards

This study was conducted and reported in accordance with the Consolidated Standards of Reporting Trials (CONSORT) 2010 guidelines. A completed CONSORT checklist is included as an additional file.

### Study design and setting

This multicenter, randomized, controlled, single-blinded (researchers) parallel-group interventional trial was conducted from January to September 2023 across six medical spa centers in Lithuania: Gradiali (Palanga), Atostogų Parkas (Kretinga region), Eglė (Druskininkai), Draugystė (Druskininkai), Tulpė (Birštonas), and Versmė (Birštonas). The study adhered to the principles of the Declaration of Helsinki and was approved by the Kaunas Regional Research Ethics Committee (approval code: BE-2-87). The trial was registered in the ClinicalTrials.gov registry (Identifier: NCT06018649).

### Participants and sample size

Eligible participants were adults aged 18–65 years with self-reported moderate to high stress levels (stress intensity > 3 on a 10-point scale; Table 1) or suboptimal stress management scores (< 7 on a 10-point scale; Table 2). Participants assessed their perceived stress symptoms - including physical, emotional, cognitive, and behavioural components - experienced over the past month.

**Table 1:** Stress intensity scale for participant inclusion

0	1	2	3	4	5	6	7	8	9	10
No stress	Low stress			Moderate stress			High stress			Unbearable stress

Source: Developed by the research team

**Table 2:** Stress management scale for participant inclusion

0	1	2	3	4	5	6	7	8	9	10
Does not manage stress at all	Poorly			Moderately			Well			Extremely well

Source: Developed by the research team

Tables 1 and 2 present visual analog-type inclusion scales developed by the research team. Participants were asked to rate their perceived stress intensity and overall stress management effectiveness over the past month using a 0–10 scale, where higher stress or lower coping indicated eligibility for inclusion.

Research participants were also asked to reflect on their overall stress management effectiveness considering all the ways they tried to overcome stress during the past month. They were asked to mark the number which corresponds to the overall stress management.

Exclusion criteria included uncontrolled or decompensated systemic illnesses (hematologic, endocrine, rheumatologic, renal, cardiovascular, gastrointestinal, or pulmonary), active infection, malignancy, recent major surgery or trauma (within 12 months), prior balneotherapy within 3 months, pregnancy or lactation, active bleeding, and severe mental or physical conditions. 

Sampling followed a probabilistic cluster (nested) design with multi-stage, criterion-based inclusion. Sample size calculations were based on data from a previous trial [24] using G*Power software [25]. With an effect size of 0.32, 0.4, and 0.5, the required group sizes were estimated at 79, 52, and 34, respectively. To accommodate for data loss, a medium effect size of 0.4 was selected, and a target of 55 participants per group was established.

### Randomization and blinding

Participants meeting eligibility criteria were randomized into study groups following initial screening (T0) in either the Klaipeda or Druskininkai cluster. A biostatistician generated the random allocation sequence using computer software. Group assignments were concealed from the investigators conducting outcome assessments to ensure single blinding. All participants provided informed consent prior to enrolment.

Study groups included:

- 11ABT: 11-day ambulatory balneotherapy complex
- 11ABTNT: 11-day ambulatory balneotherapy plus nature therapy
- 11BTS: 11-day inpatient balneotherapy
- 11 C: 2-week control group (no treatment)

**Fig. 1:**
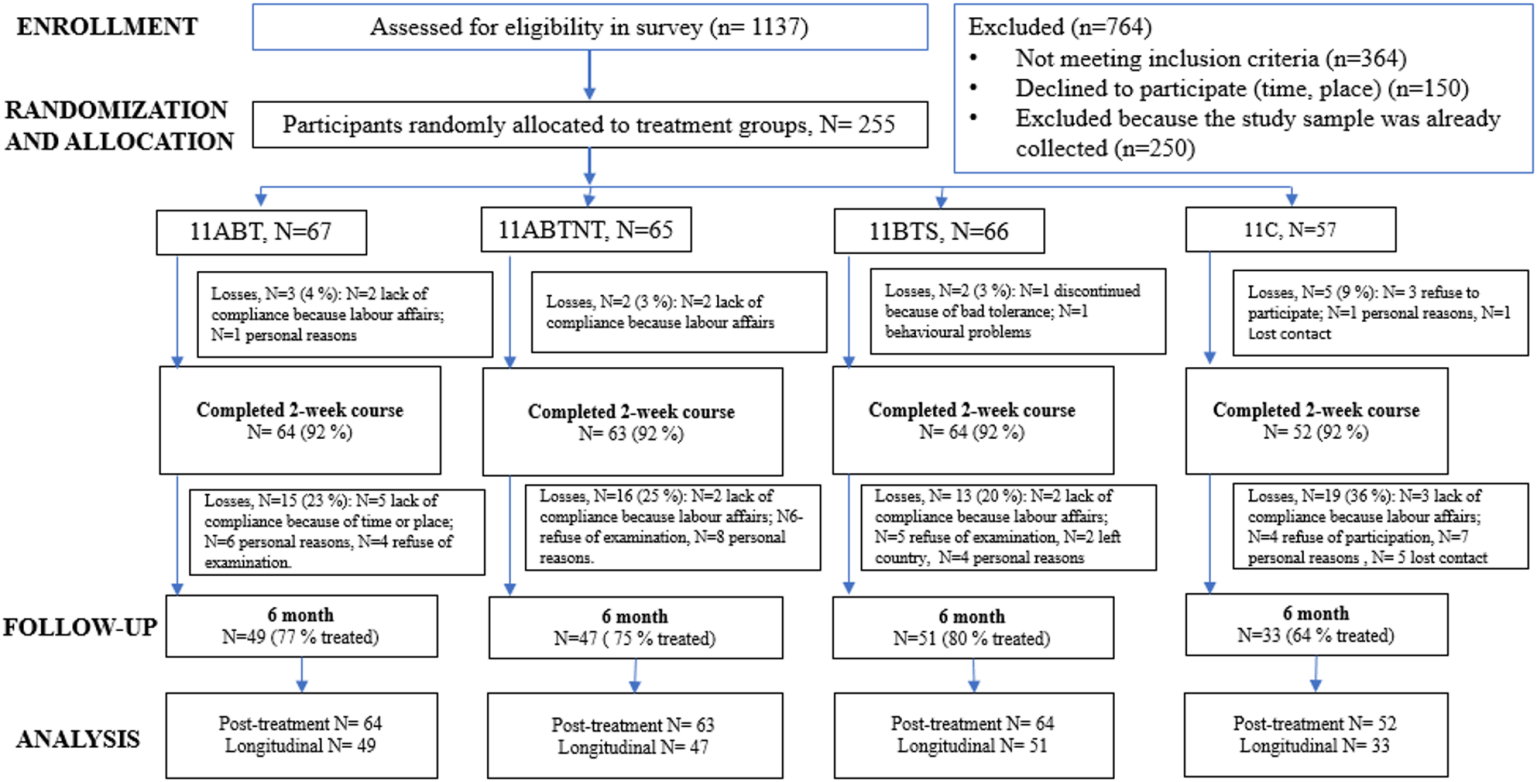
The study flow chart

Outcomes were assessed at four time points: baseline (T0), post-intervention (T1), 3-month follow-up (T2), and 6-month follow-up (T3). Of 1137 individuals screened, 764 were excluded due to not meeting inclusion criteria or declining to participate. A total of 373 participants were enrolled and randomized. After baseline assessment, 130 were excluded due to incomplete data or withdrawal, leaving 243 participants for post-treatment analysis, and 180 participants completed the 6-month follow-up and were included in the longitudinal analysis (see Fig. 1).

### Outcomes and instruments

Primary Outcomes:

- Stress intensity
- Stress management
- Depression
- Anxiety

Secondary Outcomes:

- Fatigue
- Sleep quality
- Work and social adjustment
- Cognitive functioning
- Treatment safety

Stress intensity was measured using the General Symptoms Distress Scale (GSDS), where participants rated 14 stress-related symptoms on a 10-point scale [26]. Stress management was also self-rated on a 10-point scale, with higher scores indicating better management.

The stress hormone cortisol was measured via salivary samples, collected using standardized Salivette^®^ collection devices (Sarstedt, Germany). Participants provided samples that were immediately refrigerated and sent to a certified clinical diagnostics laboratory in Germany for analysis using enzyme-linked immunosorbent assay. Cortisol values were reported in nmol/L. Salivary cortisol is a non-invasive, ecologically valid biomarker of stress-related HPA axis activity and is commonly used to assess treatment efficacy for stress-reduction interventions [13]. 

Perceived stress was evaluated using the 10-item Perceived Stress Scale (PSS-10) [27], which ranges from 0 to 40 (low: 0–13; moderate: 14–26; high: 27–40) [28].

Depression was assessed using the Center for Epidemiologic Studies Depression Scale – Revised (CESD-R), a validated 20-item self-report instrument designed to screen for symptoms of depression in line with the diagnostic criteria of the DSM-IV [29]. The CESD-R measures the frequency of depressive symptoms experienced over the past two weeks across nine symptom domains: sadness (dysphoria), loss of interest (anhedonia), appetite changes, sleep disturbances, concentration difficulties, feelings of guilt or worthlessness, fatigue, psychomotor agitation or retardation, and suicidal ideation.

Each item is rated on a 5-point Likert scale: 0 = “Not at all or less than one day”, 1 = “1–2 days”, 2 = “3–4 days”, 3 = “5–7 days”, and 4 = “Nearly every day for two weeks”.

The total score ranges from 0 to 80, with higher scores indicating more severe depressive symptoms. While a cut-off of 16 is sometimes used to identify clinically significant depressive symptoms, we analyzed CESD-R scores as a continuous variable in this study to capture changes in severity over time.

The CESD-R has been widely used in population-based and clinical studies and demonstrates strong internal consistency and construct validity.

Anxiety was measured using the 5-item short form of the Spielberger State-Trait Anxiety Inventory (STAI-5), assessing both state (STAIS) and trait (STAIT) anxiety [30]. Scores ≥ 10 for STAIS and ≥ 14 for STAIT indicate clinically relevant anxiety. The tool was used as a continuous variable.

Fatigue was evaluated using the Fatigue Assessment Scale (FAS), a 10-item tool measuring physical and mental fatigue (range: 10–50). Scores above 22 indicate significant fatigue [31, 32].

Sleep quality was assessed using the Single-Item Sleep Quality Scale (SQS), a visual analogue scale (0–10), grouped into five quality categories [33].

Functional impairment was measured using the Work and Social Adjustment Scale (WSAS), a 5-item tool assessing domains such as work, home management, and social functioning. Scores > 20 indicate moderate to severe impairment [34, 35].

The PSS-10 [36] and CESD-R [37] scales were previously validated in Lithuanian populations. Other instruments used in this study (e.g., FAS, STAI-5, WSAS, SQS) were translated from English to Lithuanian and then back-translated by an authorized professional translation office to ensure accuracy. While not all instruments have formal validation in the Lithuanian language, internal consistency was assessed during pilot testing and found acceptable for exploratory use.

Cognitive functioning (working memory, attention, speed, and visual field) was assessed using RehaCom computerized neurocognitive software [38]. Higher scores reflect better performance.

The use of sedatives, hypnotics, and antidepressants was assessed using a custom 5-point Likert scale developed by the research team for clinical self-report. Participants were asked to rate their usage frequency during the previous month on a scale from 1 (no use) to 5 (daily use). While not based on a validated instrument, the scale allowed for categorical comparisons across groups. All participants received the same instructions during baseline and follow-up assessments.

Adverse events were monitored prospectively using standardized checklists evaluating symptom frequency, severity, duration, and the need for medical treatment [39]. Treatment safety was rated on a 5-point Likert scale (1 = extremely safe; 5 = unsafe).

### Interventions

All treatment groups received identical durations and types of procedures across 11 days.

- 11ABT: Ambulatory BT complex
- 11ABTNT: Ambulatory BT complex plus nature therapy
- 11BTS: Inpatient BT complex
- 11 C: No treatment (control)

The balneotherapy complex included:

- Tap water pool with light exercises (20 min)
- Mineral or geothermal water bath at 34–36 °C (20 min)
- Sapropel mud wrapping (20 min)
- Salt therapy (25 min)

The nature therapy involved a 45-minute walk (forest or seaside), low-intensity physical and breathing exercises, multisensory stimuli (aromatherapy, natural sounds, visual input), and heliotherapy. The first session was supervised by a kinesiotherapist; remaining sessions were completed independently using provided instructions.

### Natural resources

Mineral water used in spa centers varied in total mineralization (16.75–82.445 g/L) and contained a range of macro- and microelements (e.g., Na+, K+, Ca2+, Mg2+, Cl–, SO42–, Br, Fe, Si, B). Full chemical compositions by center are detailed in Table 3.

**Table 3:** The characteristics of mineral waters used in study centers

Centers	Total mineralization, g/l	Dominant macroions, mg/l						pH	CO_2_ balanced mg/l	Other elements
		Cations			Anions					
		Na^+^+K^+^	Ca^2+^	Mg^2+^	Cl^−^	HCO_3_^−^	SO_4_^2−^			
1	55,221	15,579	3453	1387	32,075	46,6	2642	6,53	24,9	Br 81.8, K 133, Fe 18, Si 6,1, B 1,13
2	35,837	9089	2800	1166	21,000	92,5	1675	6,84	24,2	Br 52.5, K 73, Fe 6,42, Si 8,2, B 0,54
3	21,667	4304	1969	1259	12,350	110	1673	7,49	6,45	Br 46.2, K 93,9, Fe 0,31, Si 8,3, B 1,71
4	22,213	4108	2080	1400	12,500	29,9	2046	7,3	/	Br 46, К 88.1, Fe 0.26,F 0,48
5	16,75	4431	1267	417	8650	119	1862	7,54	6,22	Br 47.5, K 157, Fe 0,11, Si 7,2, B 2,25
6	82,445	22,618	6302	1948	49,900	< 10	1647	5,71	32,2	Br 270, K 518, Fe 10,1, si < 2, B 7,95

Peloids used for sapropel treatments ranged in pH (6.6–7.0), humidity (70.5–96%), mineralization (38.5–20,007 mg/L), and organic content (14.32–91.96%). Detailed composition and botanical origin are presented in Table 4.

**Table 4:** The characteristics of peloids used in the study centers

Parameters/ Centers	1	2	3	4	5	6
In native material:						
pH 1:5	7	8,3	6,9	6,4	6,6	6,7
Humidity %	79,11	70,51	94,95	75,49	94,04	96
Particulate pollution (> 2 mm)%	2,5	4	2,8	/	2,8	0,8
LT > 0,5 mm < 5%						
Hydriocarbons (HCO3) mg/l	244	366	107	185	229	214
Total mineralization, mg/l	20,007	1780	53	50	141	38,5
Dry residue, mg/l	24,140	2425	385	385	347	238
Chlorids (Cl) mg/l	10,103	49,6	21,3	42	10,6	7,1
Botanical composition						
Sedges %	10	30	10	/	20	15
Reeds %	80	60	80	/	70	75
Other plants %	10	10	10	/	10	10
In dry material:						
Ashenness %	29,27	85,68	8,04	21,36	8,83	18,08
Organic material %	70,73	14,32	91,96	78,54	91,17	81,92
LT > 50 D, 10–50 S						
Degree of fragmentation r %	81,36	100	76,07	73	79,44	75,91
Total nitrogen (N)%	2,15	0,95	7,92	/	5,7	4,05
Nitrogen (N-NO3 + N-NO2 mg/kg	33,65	2,29	1,64	/	2,55	1028
Total phosphorus (P) %	0,12	0,013	0,044	/	0,042	1,5
Total potassium (K) %	0,04	0,07	0,07	5,4 mg/l	0,06	2,25
Total sodium (Na) %	3,07	0,03	0,06	14	0,04	0,75
Total calcium (Ca) mg/kg	43,375	319,333	13,150	70	13,800	30,427
Total magnesium (Mg) mg/kg	3979	4833	942	15	954	1409
Silicea (SiO2) %	3,35	10,47	4,07	/	4,32	11,51
Total suplhur (S) %	0,55	0,24	0,89	З1,5 sulf	0,87	2,88
Humic acid content %	2,25	1,22	14,65	/	6,54	28,25
Fulvic acid content %	17,9	0,98	11,68	/	14,09	3,25
Iron (Fe) mg/kg	20,800	7450	2203	0,62 mg/l	2189	6335
Manganese (Mn) mg/kg	194	394	123	/	119	151

### Statistical analysis

Descriptive statistics were presented as means and standard deviations (SD), with 95% confidence intervals (CI) illustrated graphically. Group comparisons were performed using independent two-tailed t-tests for continuous variables and Chi-square or z-tests for categorical variables.

Between-group differences were assessed using one-way ANOVA with Tukey’s HSD post-hoc tests. Repeated measures were analyzed using the General Linear Model (GLM) for dependent variables measured across timepoints (T0–T3), with Bonferroni correction applied for multiple comparisons. If assumptions of normality were not met, the Friedman non-parametric test was used.

Effect sizes (Cohen’s d) were calculated to interpret the magnitude of treatment effects. A p-value of < 0.05 was considered statistically significant. All statistical analyses were conducted using SPSS version 28.0 (SPSS Inc., Chicago, IL, USA).

## Results

### Baseline characteristics of participants

The baseline sociodemographic and clinical characteristics of the study participants are summarized in Table 5. There were no statistically significant differences among groups in age, gender, education level, employment status, or baseline stress parameters, confirming successful randomization.

**Table 5:** Sociodemographic and clinical characteristics of study participants across intervention and control groups

	11ABT, *N* = 64	11ABTNT, *N* = 63	11BTS, *N* = 64	11 C, *N* = 52	*p*- value
Age, years, mean (SD)	48.8 (11.5)	46.3 (10.1)	48.8 (10.5)	45.8 (11.0)	0.271
Gender					0.086 ^b^
Woman, N (%)	56 (87.3)	42 (66.1)	46 (71.9)	39 (75.5)	
Man, N (%)	8 (12.7)	21 (33.9)	18 (28.1)	13 (24.5)	
Education					0.052 ^b^
Secondary, N (%)	8 (14.5)	10 (16.4)	2 (3.2)	8 (16.3)	
Higher education, N (%)	7 (12.7)	7 (11.5)	8 (12.9)	3 (6.1)	
High college education, N (%)	7 (12.7)	15 (24.6)	13 (21)	5 (10.2)	
University, N (%)	33 (60)	29 (47.5)	38 (61.3)	33 (67.3)	
Marital status					0.156 ^b^
Married, N (%)	36 (65.5)	42 (68.9)	36 (59)	37 (75.5)	
Single	9 (16.4)	4 (6.6)	6 (9.8)	8 (16.3)	
Divorced	7 (12.7)	11 (18)	16 (26.2)	3 (6.1)	
Widower	3 (5.5)	4 (6.6)	3 (4.9)	1 (2)	
Living place, city, N (%)	41 (74.5)	37 (60.7)	40 (64.5)	36 (73.5)	0.176 ^b^
Working area					0.449 ^b^
Clerck/officer, N (%)	25 (46.3)	27 (44.3)	36 (58)	22 (44.9)	
Service area, N (%)	20 (37)	22 (36.1)	12 (19.4)	21 (42.9)	
Industrial area, N (%)	2 (3.7)	5 (8.2)	8 (12.9)	3 (6.1)	
Others, N (%)	7 (13)	7 (11.4)	6 (9.7)	3 (6.1)	
Stress intensity, VAS, mean (SD)	6.31 (1.8)	6.85 (2.0)	6.83 (1.9)	6.5 (1.8)	0.333
Stress management, VAS, mean (SD)	5.25 (1.6)	5.44 (1.9)	5.41 (1.8)	5.38 (1.7)	0.932
Ill with Covid-19, N (%)	39 (70.9)	51 (82.2)	45 (72.6)	38 (77.6)	0.523 ^b^
Post-Covid condition	17 (30.90	19 (30.2)	23 (37.1)	14 (28.6)	0.810 ^b^

a ANOVA test with Bonferroni correction, Pearson chi-squared (b) test, and -test, between proportions from each other

### Short-term treatment effects on stress and related outcomes

The short-term effects of the intervention on psychological and functional outcomes are summarized in Table 6. All three treatment groups—ambulatory balneotherapy (11ABT), ambulatory balneotherapy plus nature therapy (11ABTNT), and inpatient balneotherapy (11BTS)—demonstrated statistically significant improvements across most outcomes when compared to baseline (*p* < 0.05 to *p* < 0.001), whereas the control group (11 C) showed minimal or no meaningful changes.

**Table 6:** Pre- and post-treatment changes in key outcomes across study groups

Outcome	11ABT Δ Mean (SD)	11ABT *p*-value	11ABTNT Δ Mean (SD)	11ABTNT *p*-value	11BTS Δ Mean (SD)	11BTS *p*-value	11 C Δ Mean (SD)	11 C *p*-value
Salivary cortisol	-0.72 (1.58)	**0.009**	-0.87 (1.87)	**0.006**	-0.64 (2.01)	0.117	+ 0.11 (1.89)	0.748
Stress intensity	-2.60 (2.05)	**< 0.001**	-2.89 (2.15)	**< 0.001**	-2.57 (1.43)	**< 0.001**	-1.18 (2.09)	**< 0.001**
Stress management	+ 1.11 (2.17)	**0.002**	+ 0.98 (2.45)	**0.012**	+ 1.89 (1.90)	**< 0.001**	-0.16 (1.69)	0.516
PSS-10	-3.15 (7.57)	**< 0.001**	-2.76 (7.52)	**0.002**	-3.97 (4.69)	**< 0.001**	-1.35 (5.18)	**0.002**
Fatigue (FAS)	-4.13 (8.01)	**< 0.001**	-5.14 (5.99)	**< 0.001**	-4.75 (5.64)	**< 0.001**	+ 0.22 (6.39)	0.775
State anxiety (STAIS)	-2.64 (4.16)	**< 0.001**	-2.31 (4.53)	**< 0.001**	-2.12 (2.71)	**< 0.001**	-0.45 (2.24)	0.268
Trait anxiety (STAIT)	-3.47 (4.14)	**< 0.001**	-2.57 (3.93)	**< 0.001**	-3.85 (3.21)	**< 0.001**	-0.29 (3.07)	0.342
Depression (CESD-R)	-12.49 (14.44)	**< 0.001**	-10.68 (14.66)	**< 0.001**	-10.93 (9.70)	**< 0.001**	-1.275 (10.80)	0.253
Sleep quality	+ 1.98 (1.63)	**< 0.001**	+ 2.25 (2.87)	**< 0.001**	+ 2.25 (1.95)	**< 0.001**	-0.14 (1.67)	0.571
Work/social functioning (WSAS)	-2.80 (11.99)	**0.023**	-8.43 (9.83)	**< 0.001**	-7.84 (7.93)	**< 0.001**	-0.45 (10.43)	0.641
Integrative outcomes	+ 1.91 (1.63)	**< 0.001**	+ 3.04 (2.06)	**< 0.001**	+ 1.35 (1.95)	**< 0.001**	-0.07 (1.46)	0.724

△Mean (SD) - change from baseline to post-treatment; p-values indicate within-group significance. Statistically significant changes (*p* < 0.05) are highlighted in bold. Effect sizes and confidence intervals are presented in Supplementary Table

Salivary cortisol levels decreased significantly in both the 11ABT and 11ABTNT groups, indicating a physiological stress reduction. All treatment groups showed substantial reductions in self-reported stress intensity (effect sizes ranging from 0.78 to 1.32) and perceived stress scale (PSS-10) scores. Notably, stress management ability significantly improved in the 11BTS and 11ABT groups, but not in the control group.

Depression symptoms (CESD-R) and anxiety levels (both state and trait) decreased significantly in all intervention groups, with large effect sizes particularly evident in the inpatient group (e.g., STAIT d = 1.49). Fatigue levels (FAS) also declined significantly across all treatment groups but remained unchanged in the control group.

Functional impairment, as measured by the Work and Social Adjustment Scale (WSAS), showed marked improvement in the treatment groups (*p* < 0.001), with the most prominent reduction seen in 11ABTNT. Sleep quality improved substantially in all treatment arms (*p* < 0.001), with the 11BTS group showing the greatest effect size (d = − 1.47). Similarly, integrative well-being scores significantly increased in all treatment groups but remained unchanged in the control group.

No statistically significant changes were observed in cognitive domains such as working memory, processing speed, attention, or visual field across any of the groups. Likewise, the use of antidepressants remained stable during the study period. A small but statistically significant reduction in the use of sedatives and hypnotics was observed in the 11ABTNT group only (*p* < 0.001).

Overall, the data indicate that a short-term course of balneotherapy—especially when delivered in an inpatient setting—can produce rapid and clinically meaningful improvements in stress-related mental health and functioning.

**Fig. 2:**
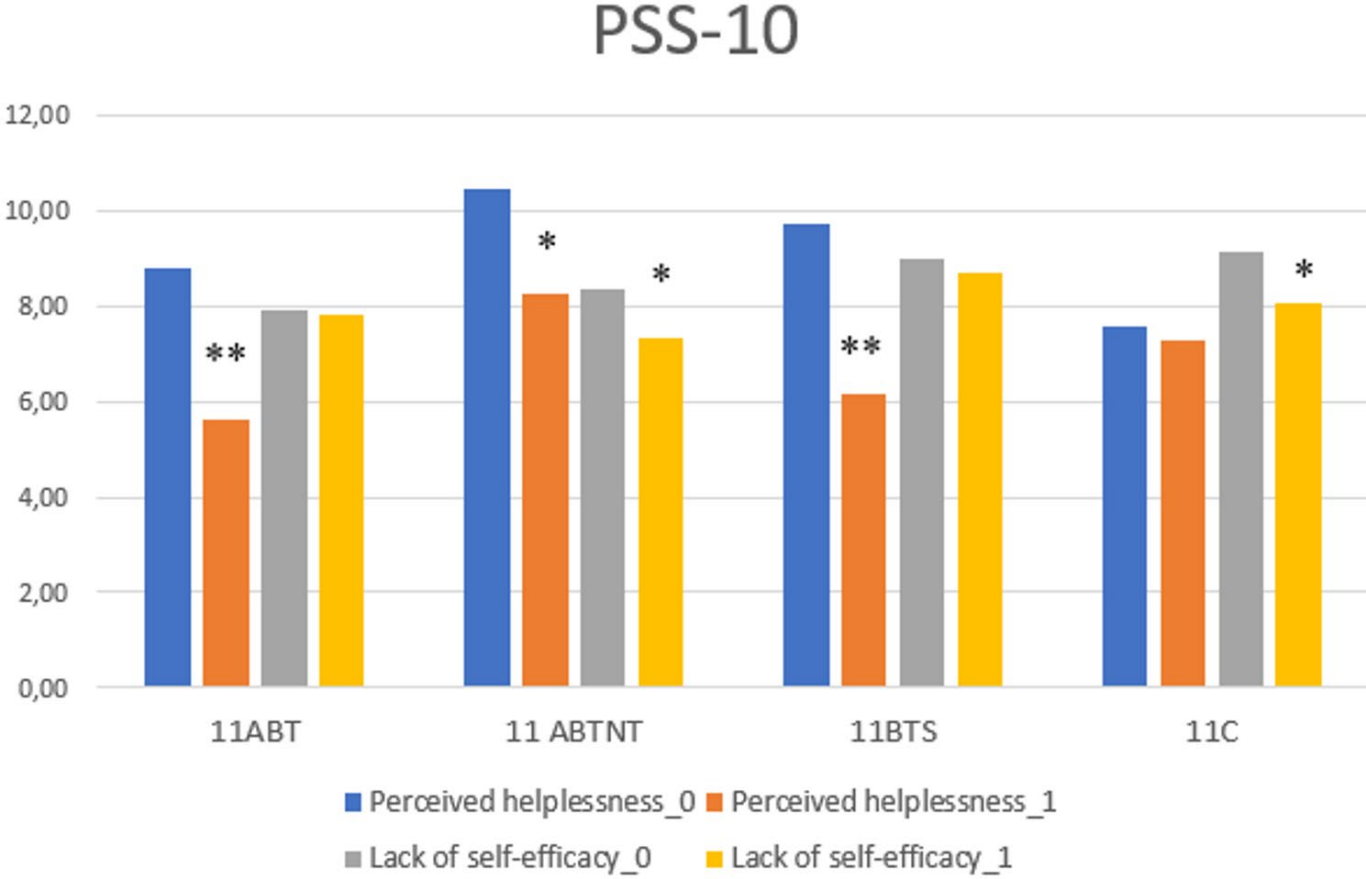
The change of perceived stress subscales in study groups. ** *p* < 0.001; * *p* < 0.05

Further analysis of the PSS-10 subscales revealed a significant reduction in perceived helplessness across all treatment groups, while a significant improvement in perceived self-efficacy was observed exclusively in the 11ABTNT group (Fig. 2).

**Fig. 3:**
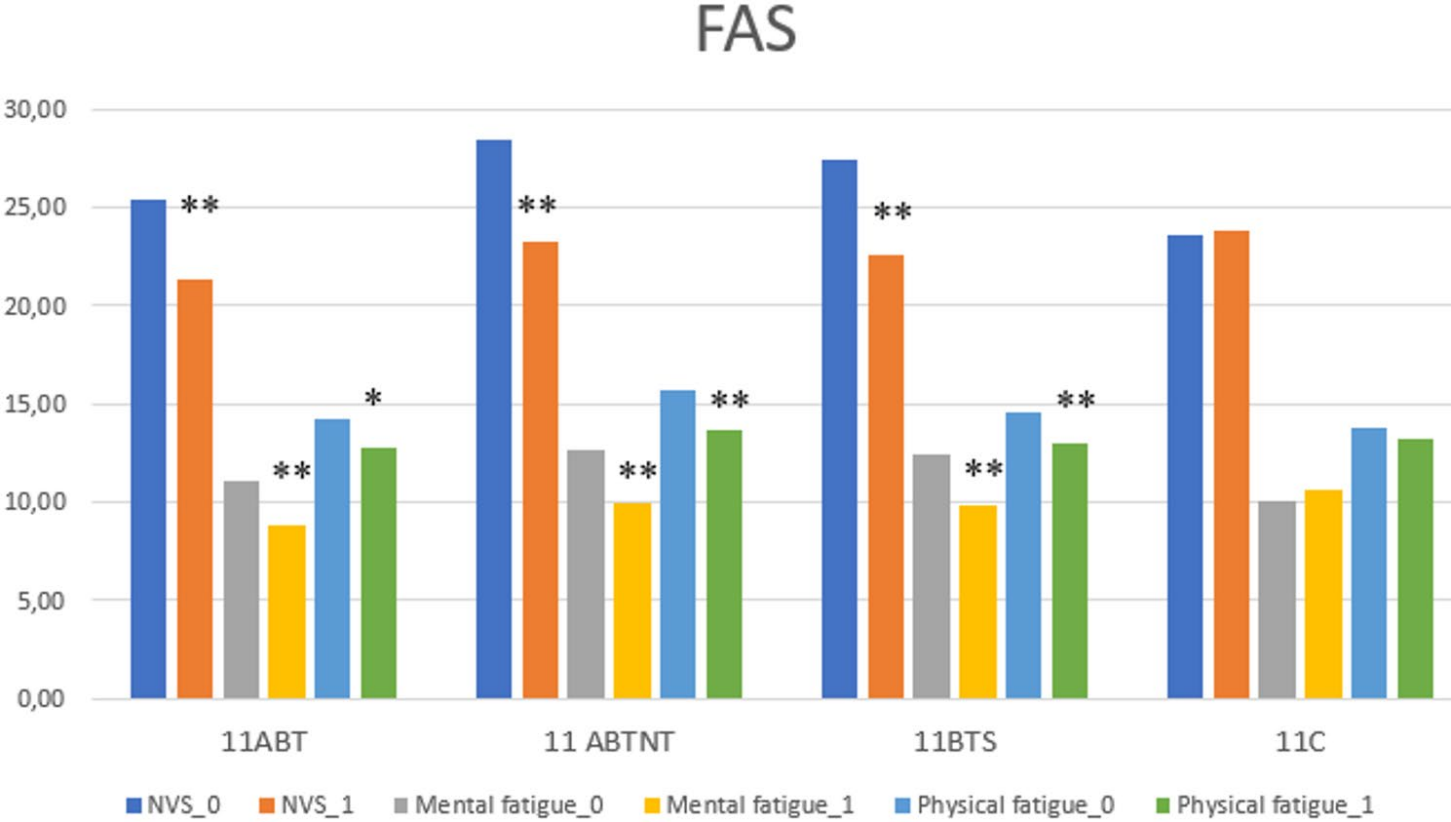
The change of fatigue subscales in study groups. ** *p* < 0.001; * *p* < 0.05

At baseline, all study groups exhibited mild levels of fatigue, with the lowest fatigue scores recorded in the control group. Following the intervention, significant improvements in fatigue were observed across all treatment groups (*p* < 0.001), with reductions ranging from 17% to 19% and corresponding medium to large effect sizes. Notably, fatigue levels in the 11ABT group reached within the normative range post-treatment. Improvements were evident in both physical and mental fatigue subscales (Fig. 3).

Baseline anxiety levels across all study groups were below the threshold for clinically significant anxiety, with the control group exhibiting the lowest initial scores. Nonetheless, all treatment groups demonstrated significant reductions in both state and trait anxiety following the intervention (*p* < 0.001), with medium to very large effect sizes. Mean anxiety scores were reduced by up to 30% in the 11ABT and 11BTS groups.

**Fig. 4:**
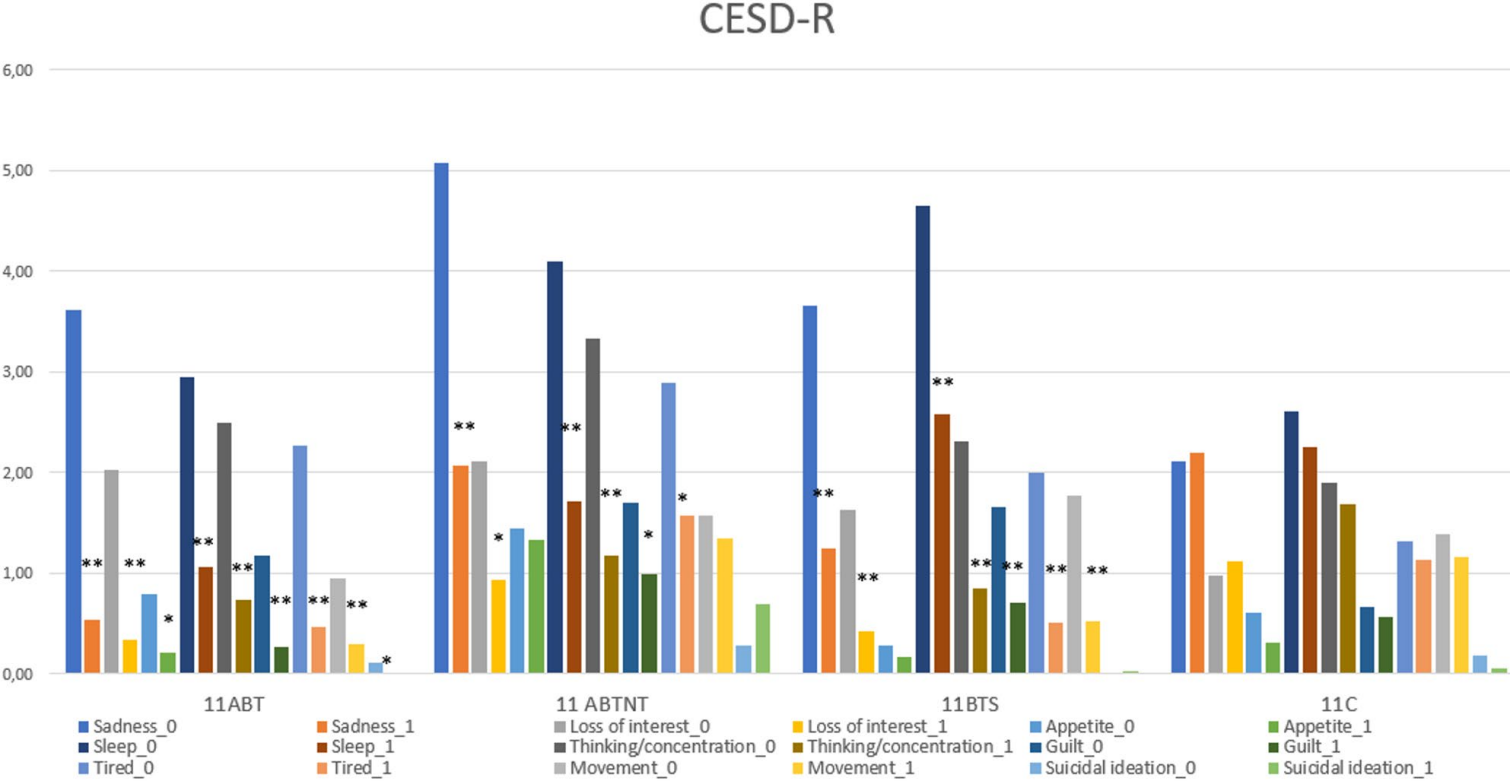
The change in depressive symptoms subscales in study groups. ** *p* < 0.001; * *p* < 0.05

Subthreshold depressive symptoms were identified in all groups except the control. Post-treatment, depression levels in all intervention groups decreased to below clinical relevance, with effect sizes ranging from medium to very large. The most pronounced reduction was observed in the 11ABT group, where depression scores decreased by up to 76%. Subscale analysis revealed significant improvements in all nine CESD-R domains in the 11ABT group, six domains in 11ABTNT, and seven in 11BTS. The least responsive subscales were suicidal ideation, appetite, and movement (Fig. 4).

Subgroup analyses based on baseline trait anxiety levels were not performed, as the study was not powered or stratified for this purpose.

At baseline, participants in all groups exhibited moderate impairment in work and social functioning. Following the intervention, significant improvements were observed in all treatment groups (*p* < 0.001), with gains of up to 49%. The largest effect was noted in the 11BTS group, where functional impairment scores improved to a subclinical level.

Sleep quality also improved significantly across all treatment groups, with the most substantial change observed in the 11BTS group, where scores increased by 49%, corresponding to a very large effect size.

Integrative outcomes—which reflect overall physical, emotional, and social well-being—showed statistically significant improvements in all intervention arms, with the greatest gain of up to 61% recorded in the 11ABTNT group (very large effect).

A significant reduction in the use of sedatives and hypnotics was observed only in the 11ABTNT group, with a 16% decrease (medium effect size).

In contrast, the control group demonstrated statistically significant improvement in only two outcomes: stress intensity (23% reduction; medium effect size) and perceived stress (8% reduction; small effect), limited to the self-efficacy subscale of the PSS-10. No meaningful changes were observed in sleep, fatigue, anxiety, depression, functional adaptation, or integrative well-being within the control group.

### Long-term effects of treatment on stress and related conditions

**Fig. 5:**
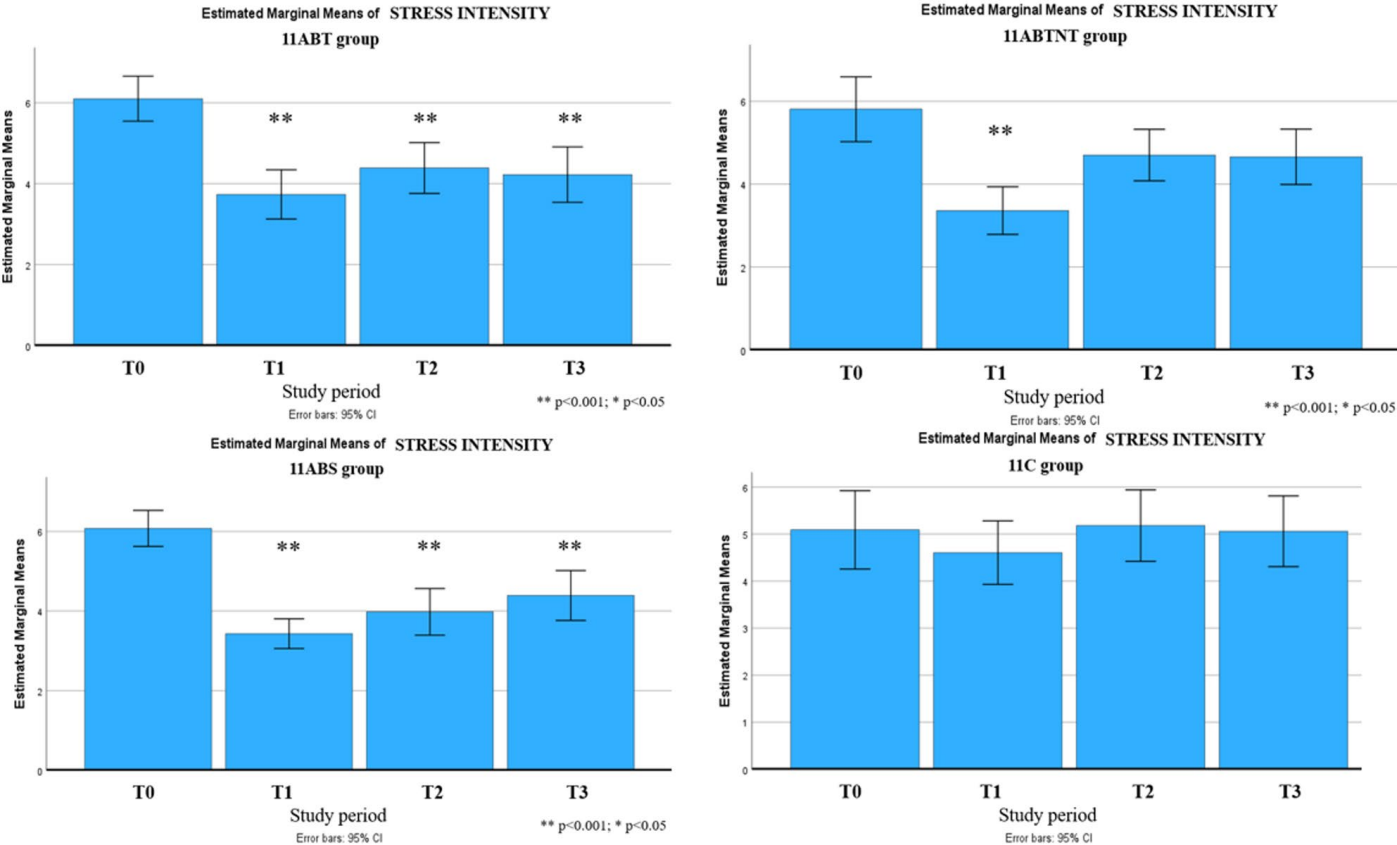
Changes in stress intensity during the follow-up period

**Fig. 6:**
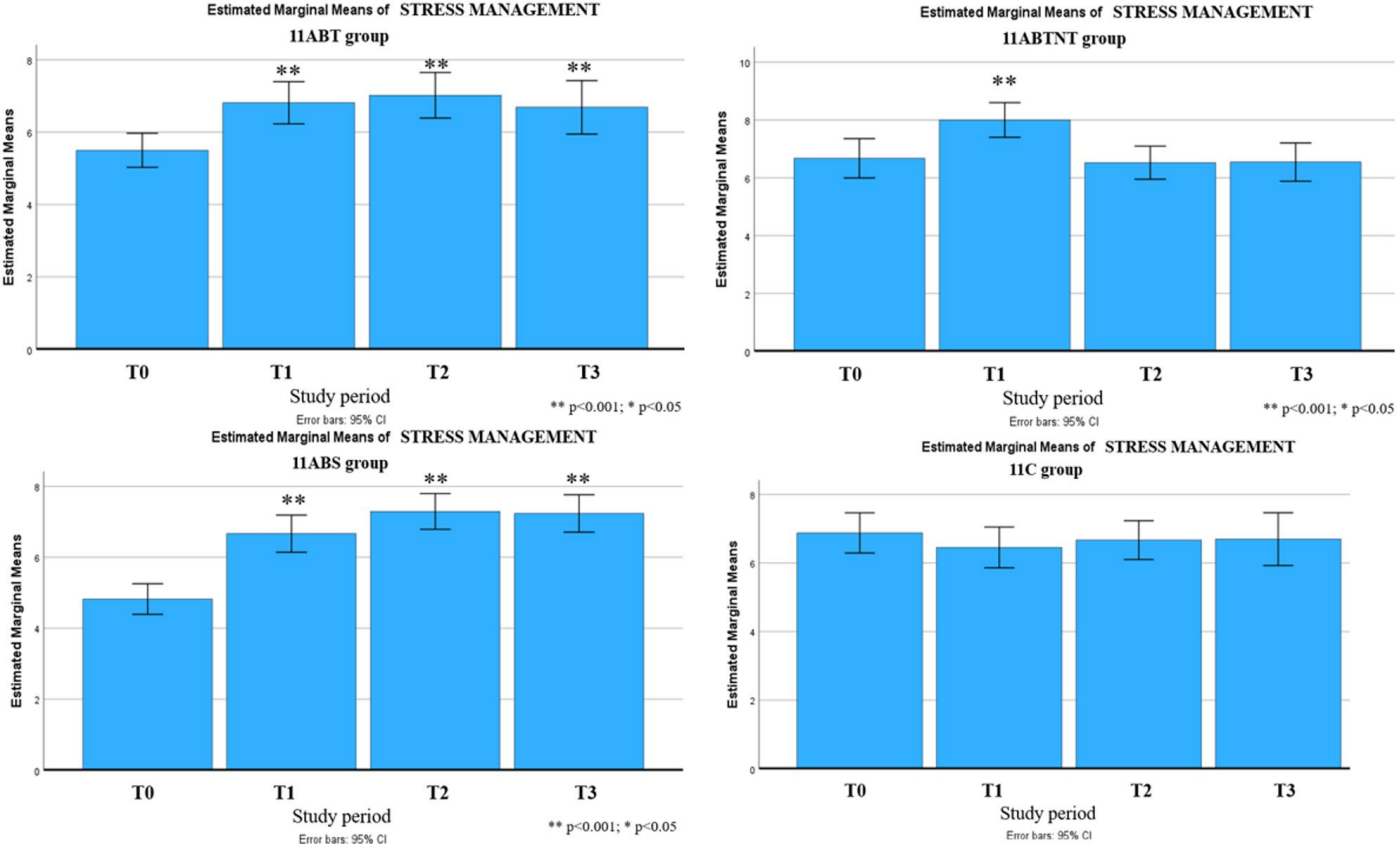
Changes in stress management during the follow-up period

**Fig. 7:**
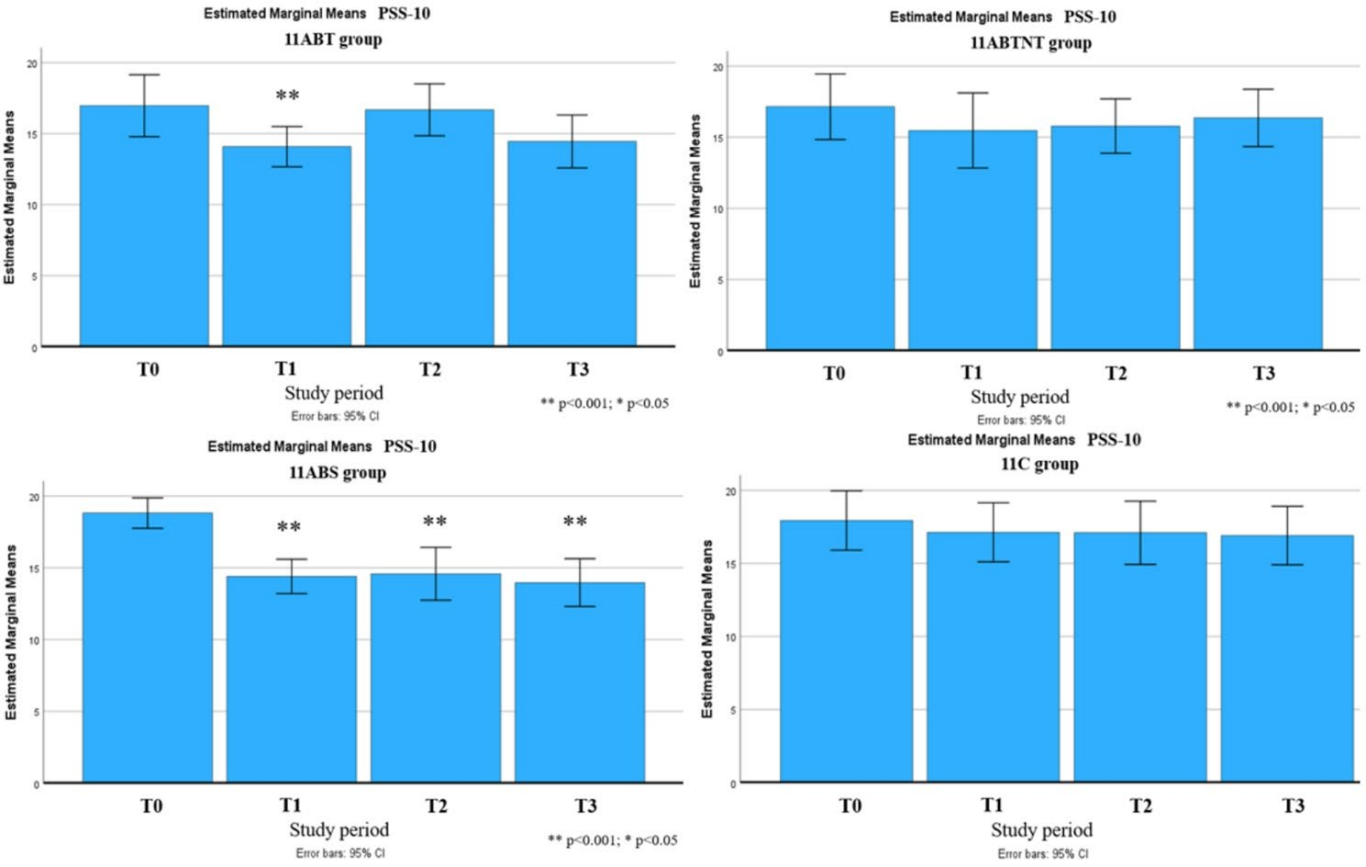
Perceived stress trajectories across groups during the follow-up period

**Fig. 8:**
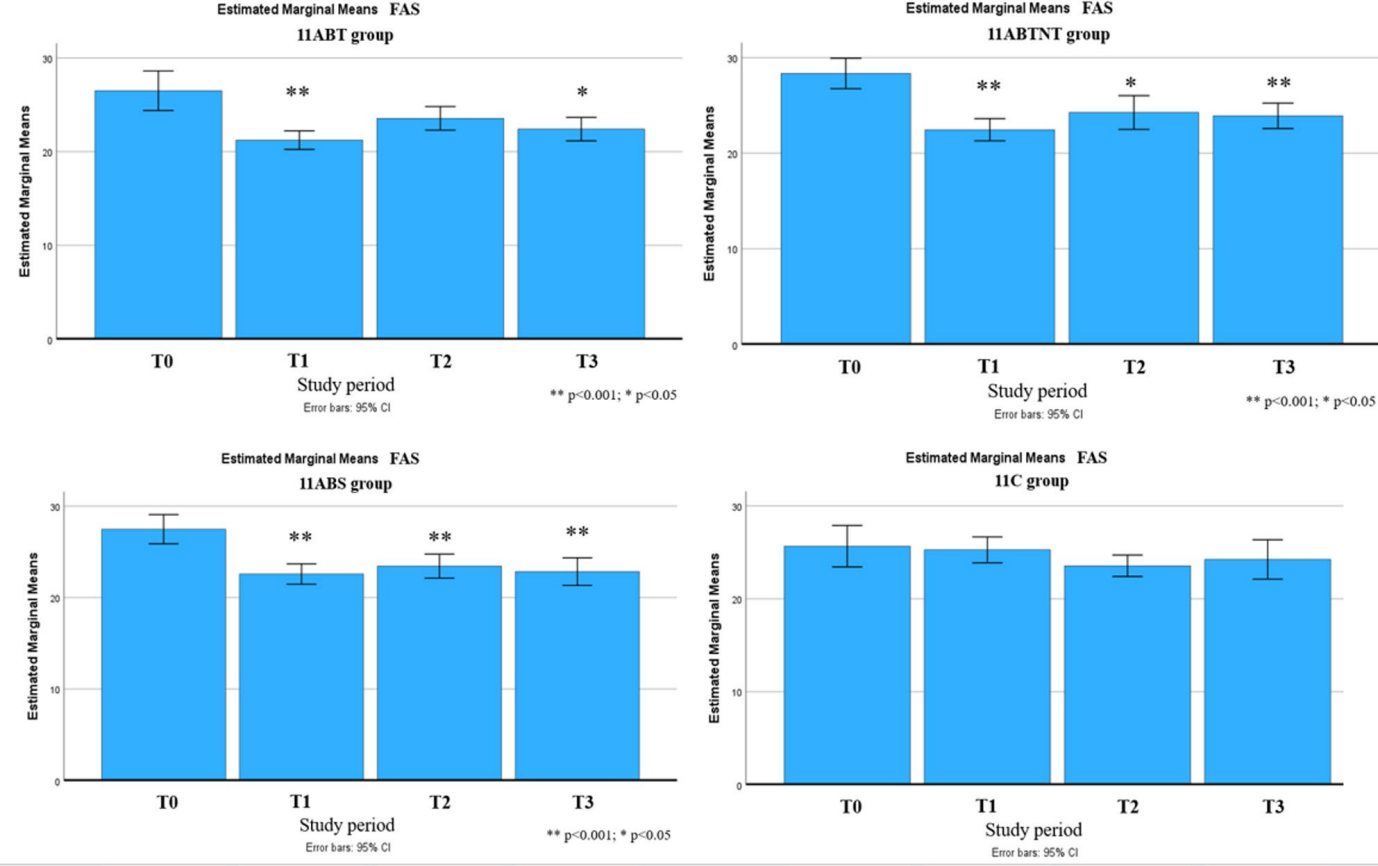
Fatigue Assessment Scale changes during the follow-up period

**Fig. 9:**
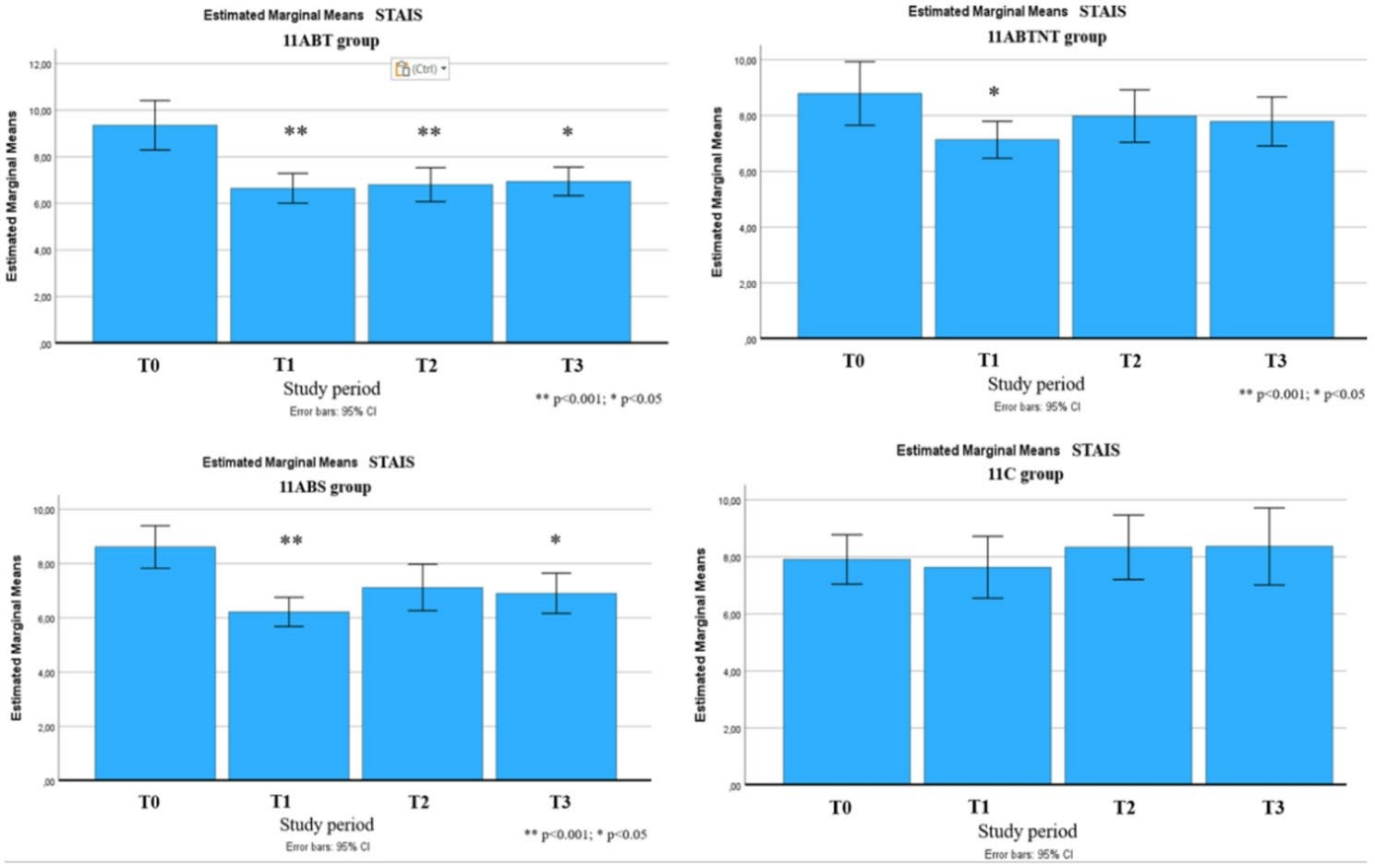
Changes in state anxiety during the follow-up period

**Fig. 10:**
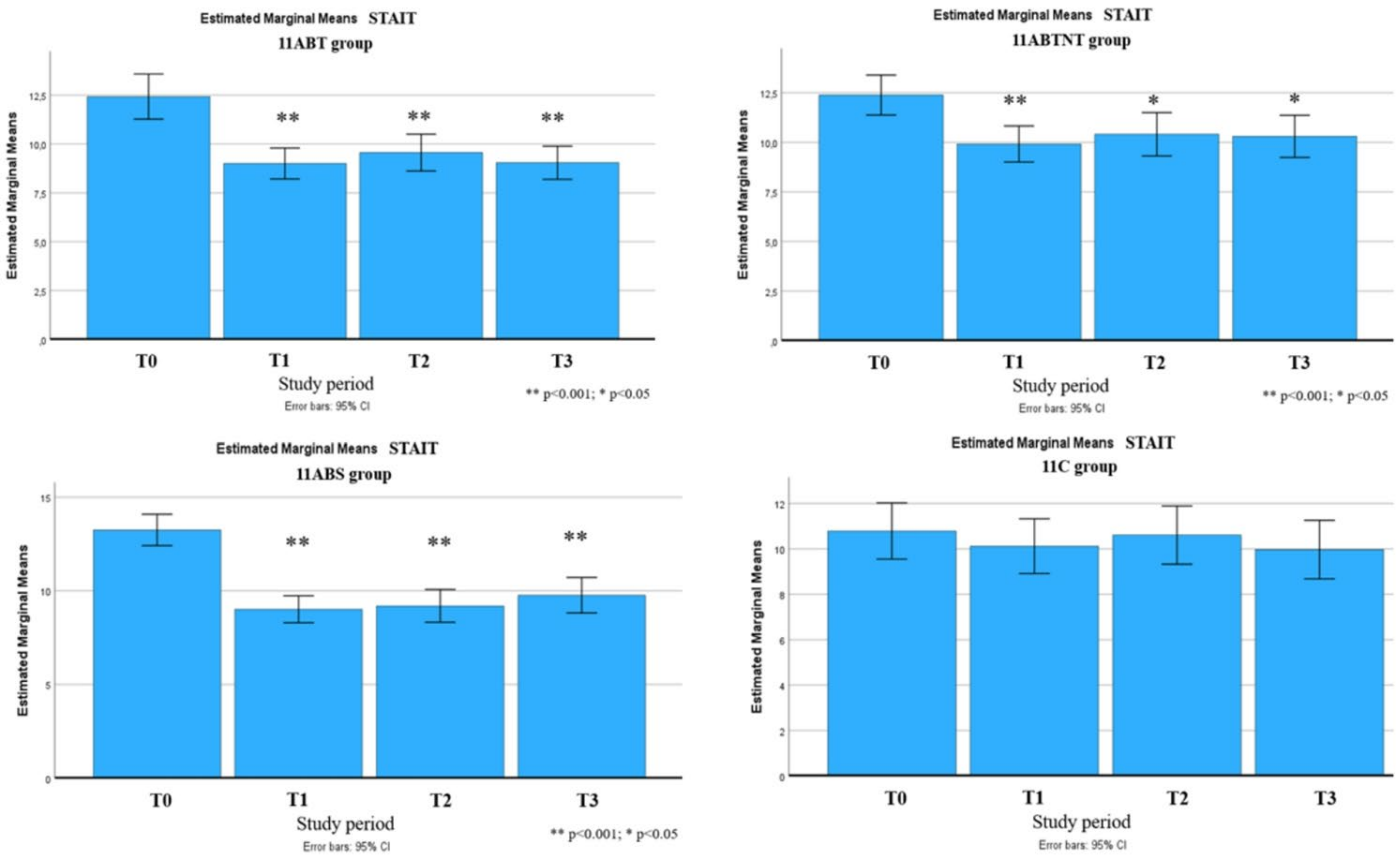
Changes in trait anxiety during the follow-up period

**Fig. 11:**
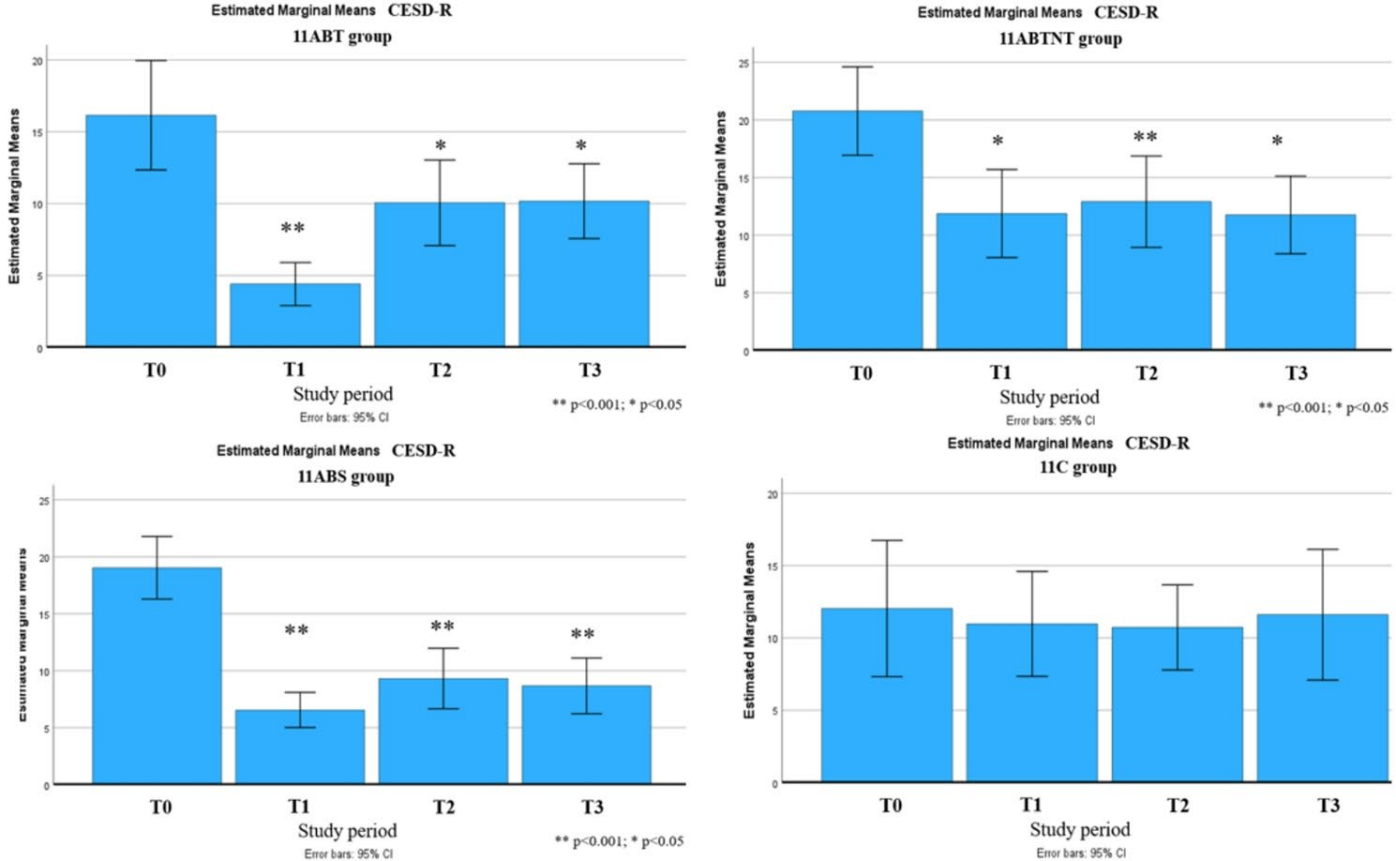
Changes in depressive symptoms during the follow-up period

**Fig. 12:**
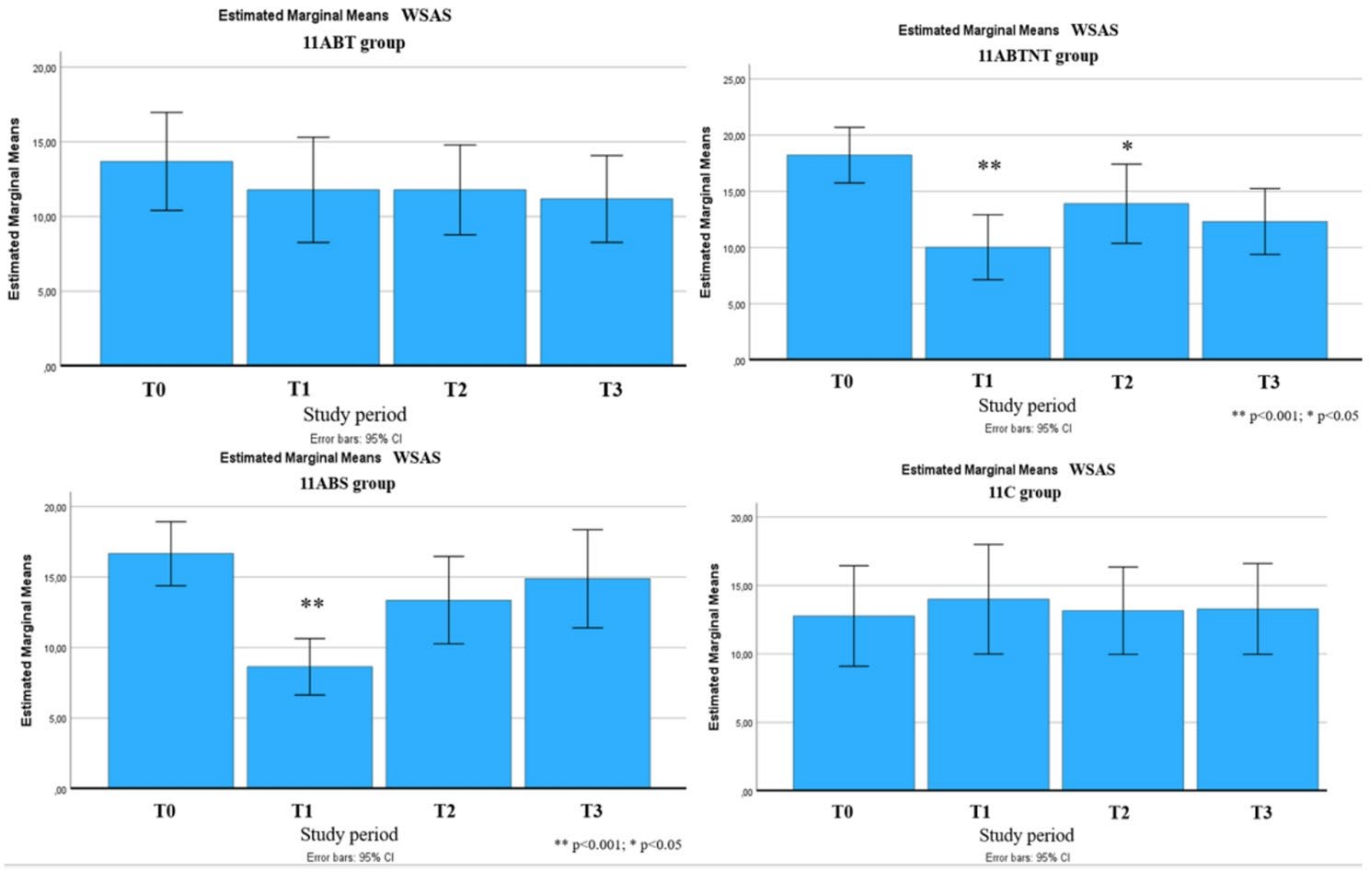
Work and Social Adjustment Scale changes during the follow-up period

**Fig. 13:**
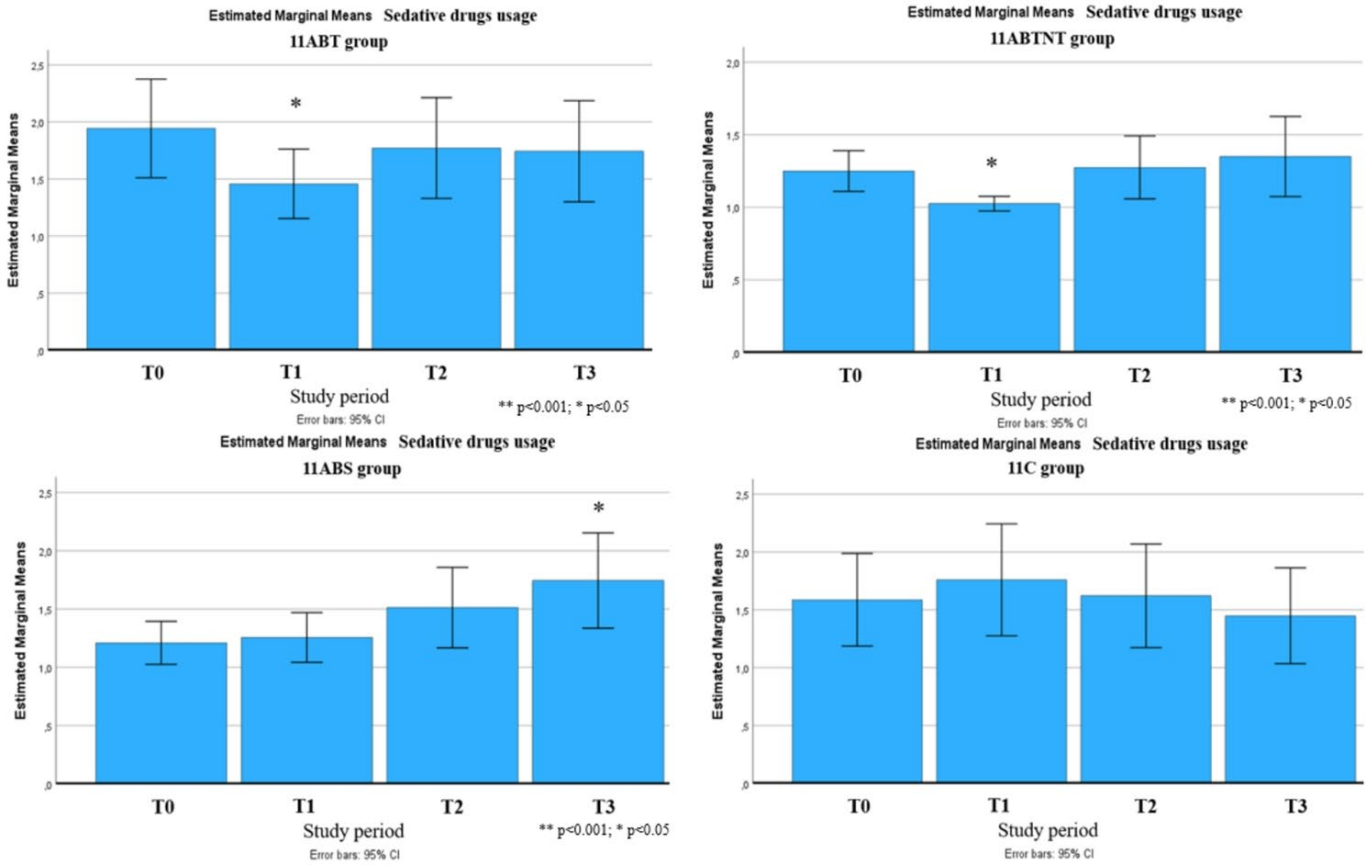
Changes in sedative usage during the follow-up period

Significant long-term improvements were observed across multiple outcomes up to six months following the intervention. These included reductions in stress intensity (up to 31% in the 11ABT group) and improvements in stress management (up to 50% in the 11BTS group), fatigue (up to 17% in 11BTS), state and trait anxiety (both up to 27% in 11ABT), depression (up to 54% in 11BTS), functional adaptation (32% in 11ABTNT), sleep quality (up to 67% in 11BTS), and integrative well-being (up to 40% in 11ABT). The most pronounced and sustained changes were observed in the inpatient treatment group (11BTS) (Figs. 5, 6, 7, 8, 9, 10, 11, 12 and 13).

Analysis of treatment durability revealed that:

- 11ABT (ambulatory balneotherapy) maintained long-term effects on stress intensity and management, anxiety, depression (particularly the loss of interest subscale), sleep quality, and integrative outcomes.
- 11ABTNT (ambulatory balneotherapy + nature therapy) sustained improvements in fatigue (both physical and mental), trait anxiety, depression (notably sadness, sleep, and thinking subscales), sleep quality, and integrative well-being.
- 11BTS (inpatient balneotherapy) showed the broadest and most enduring benefits, with lasting effects in stress intensity and management, perceived stress (lack of efficacy subscale), fatigue (mental subscale), trait anxiety, and depression (including sadness, loss of interest, sleep, thinking, guilt, tiredness, and movement subscales), as well as sleep quality and integrative outcomes.

No significant long-term changes were detected in cognitive functioning or in the use of sedatives, hypnotics, or antidepressants. Additionally, no sustained improvements were observed in the control group during the follow-up period.

While cognitive function was included as an exploratory outcome, no statistically significant improvements were observed across groups in working memory, attention, or visual field performance. Although a positive directional trend was noted in the inpatient group, the short treatment duration (11 days) may have limited the likelihood of detecting significant cognitive change. These results are discussed further in the Discussion section.

### Treatment safety

The overall safety profile of the intervention was favourable. A total of 88% of participants rated the treatment procedures as extremely safe, 10% as safe, and 2% as neither safe nor unsafe.

Adverse reactions were mild and predominantly transient. Reported adverse events included:

- Mineral water therapy: 15% of participants experienced reactions such as skin itching, dryness, or redness. Of these, 69% were classified as rare, 99% as mild, and 94% resolved immediately after the procedure. In 3% of cases, symptoms lasted longer than a few days, and two participants required additional medical treatment (for elevated blood pressure and heart rate).
- Pelotherapy: 7% of participants reported increased pulse, skin irritation, or rash. In 47% of cases, reactions were rare, and in 96%, they resolved immediately. Only 1% persisted beyond several days, and three participants required additional skin-related treatment.
- Halotherapy: 6% of participants reported mild symptoms such as dry mouth, thirst, or cough. These were rare in 98% of cases and resolved immediately in 99%. No additional treatment was required.

There were no serious adverse events. One participant in the 11BTS group discontinued participation due to ECG changes detected at baseline. In the 11ABTNT group, water treatment was temporarily suspended for three days in one case due to a mild adverse reaction.

## Discussion

This study demonstrated a significant positive effect of a balneotherapy (BT) complex on stress and a range of stress-related conditions. Both quantitative and qualitative improvements were observed across key indicators such as perceived stress (PSS), fatigue, depression, and functional impairment (WSAS). The most notable changes included reductions of 76% in depression, 61% in integrative well-being, 49% in adaptation and sleep quality, 46% in stress intensity, and 38% in stress management. Anxiety was reduced by 30%, salivary cortisol by 25%, perceived stress by 21%, fatigue by 19%, and sedative use by 16%. The largest effect sizes were found in the inpatient treatment group. Importantly, the beneficial effects persisted for up to six months, including reductions of 31% in stress intensity, 26% in stress level, 17% in fatigue, 27% in anxiety, 54% in depression, and improvements of 50% in stress management, 32% in work and social adaptation, 67% in sleep, and 40% in integrative outcomes.

Although rapid change in mental health may seem unlikely, neuroscience research has shown that the amygdala and prefrontal cortex—regions involved in fear, emotion regulation, cognition, and behavioral control—are capable of reversible structural plasticity in adults [9]. Balneotherapy and pelotherapy represent natural, non-invasive therapies that utilize mineral-rich water and therapeutic mud to support physical and psychological health. These interventions are known to promote relaxation and improve well-being, potentially alleviating symptoms of stress and mood disorders [40]. Immersion in warm, mineral-rich thermal water may enhance psychological well-being through a combination of mechanical buoyancy, thermal effects, and mineral absorption [41]. Similarly, peloid (mud) therapy has been associated with reductions in stress and mood symptoms, likely through its thermal and sensory effects on the nervous system [16, 52]. The present findings confirm that the BT complex led to reductions in stress intensity, salivary cortisol, and perceived stress levels, while also improving stress management. Comparable results have been reported in studies comparing bathing versus showering, where bathing had greater benefits for stress, fatigue, and pain reduction, as well as self-reported health [41]. Other trials have documented the subjective stress-relieving effects of BT [42], including reductions in salivary cortisol following short-term spa therapy [43]. A systematic review from 2018 further supported BT’s capacity to reduce cortisol levels and enhance stress resilience. Cortisol changes following BT suggest modulation of the hypothalamic-pituitary-adrenal axis, even in healthy individuals [22].

There is also a documented link between stress and cognitive impairment. The EPIC-Norfolk study reported that higher perceived stress and stressful life events were associated with lower global cognitive scores [44], and acute stress has been linked to reduced performance in attention-related tasks [45]. We hypothesize that reducing stress through BT could contribute to cognitive benefits, potentially through enhanced cerebral blood flow and relaxation. Previous studies suggest that BT and spa therapies can modulate stress physiology and promote neural recovery by reducing sympathetic nervous activity and improving sleep and mood [46]. However, despite observing trends toward cognitive improvement—particularly in the inpatient group—no significant changes were detected in working memory, speed, attention, or visual field. This may be attributed to the relatively short treatment duration (11 days). As cognitive outcomes were included as exploratory measures, their interpretation is limited by low statistical power and short intervention time. Future studies with longer duration and stratified cognitive baselines are warranted.

Consistent with previous literature, our results also showed improvements in fatigue and sleep. Warm water immersion has been shown to promote relaxation and reduce fatigue [47, 48]. Improved sleep quality observed in our study aligns with earlier findings suggesting that BT has calming effects on the nervous system, contributing to better rest. Mud applications may also reduce physical fatigue, further supporting improved sleep. Our participants demonstrated enhanced integrative well-being, reflecting physical, emotional, mental, and social improvements. These findings are consistent with other studies reporting improved sleep, function, and quality of life in patients undergoing spa therapy [49–52].

Reductions in depression and anxiety observed in this study have also been noted by other researchers. Several trials have shown that mineral water and peloid-based therapies reduce anxiety [53, 54] and depression [55]. Mud therapy has also been associated with improvements in pain, physical function, depression, and overall quality of life [56]. Experimental evidence has shown that peloids can modulate the central and autonomic nervous systems by influencing neurotransmitter systems (e.g., acetylcholine, adrenaline) and ion balance (e.g., Ca, Mg, K), thereby contributing to vegetative tone regulation and the resolution of stress-related symptoms [57, 58]. Furthermore, BT combined with electrotherapy and massage has been shown to increase serum serotonin in patients with degenerative lumbar spine conditions, a neurotransmitter involved in pain modulation and cognition [59]. Peloidotherapy remains a valuable treatment modality in rheumatologic care [60–63], and recent interest has expanded into its anti-aging potential [64].

While stress is an unavoidable aspect of modern life—aptly described as “the trash of modern life” by Danzae Pace—it must be managed effectively to prevent its long-term consequences. Complete elimination of stress is unrealistic, but its effects can be mitigated through both pharmacological and non-pharmacological approaches [65]. These include cognitive and behavioral interventions, lifestyle changes, physical activity, healthy diet, and relaxation practices such as mindfulness, meditation, yoga, breathing exercises, massage, reflexology, Reiki, and hydrotherapy [25, 66, 67]. The World Health Organization also promotes stress management strategies to build resilience and psychological coping skills [68].

Importantly, ill-being (e.g., stress, anxiety, depression) and well-being (e.g., positive emotions, life meaning, mental vitality) are not opposite ends of the same spectrum, but related constructs [15]. Interventions like balneotherapy, which improve both negative symptoms and positive well-being, may have a broad application in prevention and health promotion. If proven effective in larger and more diverse populations, these approaches could support brain health, reduce mental distress, and potentially delay the onset of neurodegenerative or mood disorders.

Given the growing global burden of poor mental health—especially in the post-COVID-19 context—balneotherapy offers a promising complementary approach. While individual responses vary and more rigorous research is warranted to elucidate underlying mechanisms, BT may be effectively integrated into multidisciplinary care plans for individuals with stress-related mental health conditions. Although not a substitute for conventional treatments, BT appears to provide safe, supportive symptom relief, particularly when applied within a structured, personalized therapeutic framework.

The findings of this study have important clinical and wellness implications. Given the observed improvements in stress, anxiety, depression, sleep, and overall psychological functioning—paired with the high safety profile—balneotherapy may serve as a viable complementary intervention in both clinical rehabilitation and preventive mental health care. It can be particularly beneficial for individuals experiencing mild to moderate stress-related symptoms who may not yet require pharmacological or intensive psychological treatments. Moreover, BT could be implemented in wellness programs, employee health initiatives, and integrative care settings, especially where access to traditional psychotherapy is limited or where patients prefer natural, non-invasive approaches. The incorporation of natural environments and mindfulness-like components in BT also suggests potential for enhancing emotional regulation and resilience. Future research should explore its comparative effectiveness against standard therapies and its long-term feasibility in real-world clinical settings.

### Study limitations

This study was a multicenter randomized controlled trial that evaluated the efficacy and safety of natural resource-based therapies across multiple physical and mental health parameters, with a follow-up period of six months. Although a standardized treatment protocol was developed for all participating centers, variability in the natural resources used (e.g., mineral composition of water, peloid properties), modes of application (e.g., baths vs. pools; thick vs. thin mud layers), rest durations, and participants’ daily activities may have introduced heterogeneity, limiting strict comparability across sites and treatment arms.

Another limitation relates to sample size—particularly during longitudinal follow-up, where the control group was relatively small. This reduced the statistical power to detect between-group differences and limits the generalizability of the findings. Future studies should aim for larger and more diverse populations to enhance external validity and subgroup analyses.

Participant attrition during the follow-up period also presented a challenge. As ethical regulations permitted participants to withdraw at any stage without consequence, maintaining adherence across multiple centers was difficult. This impacted the completeness of long-term data and may have introduced attrition bias.

Although a wide range of validated instruments was used to assess physical and mental outcomes, some differed from those employed in similar trials. This may hinder cross-study comparisons. Furthermore, while some instruments were fully validated in Lithuanian, others were only translated and back-translated, which may affect psychometric consistency. The use of universally standardized, culturally adapted tools would improve reliability and comparability.

The heterogeneity of participant health statuses may also have influenced treatment response, making it difficult to attribute outcomes to specific baseline characteristics or comorbidities. No stratified subgroup analyses were performed for trait anxiety or other potential effect modifiers.

Additionally, while some of the questionnaires used in this study (e.g., PSS-10, CESD-R) were validated in the Lithuanian language, other instruments—though professionally translated—were not formally psychometrically validated in the native context. This may limit the cultural sensitivity and reliability of certain measurements.

Finally, the relatively short intervention duration (11 days) provides only a limited view of long-term benefits. While positive effects persisted for six months, it remains unclear whether effects extend beyond that period. Future research should incorporate longer intervention durations and follow-up intervals. Additionally, harmonized protocols for balneotherapy implementation across centers are needed to reduce variability and support reproducibility.

While the exclusion criteria controlled for balneotherapy within three months prior to study entry, participants were asked—via informed consent—not to engage in any balneotherapy during the six-month follow-up. Although this approach aimed to isolate the effects of the intervention, future studies may consider extending the exclusion window and employing active monitoring to strengthen post-intervention control.

## Conclusions

Balneotherapy complex treatment significantly reduced levels of stress, fatigue, anxiety, and depression, while improving stress management, sleep quality, work and social functioning, and overall integrative well-being. These effects were confirmed across multiple validated outcomes, with several domains showing medium to very large effect sizes. Importantly, many benefits persisted for up to six months, particularly among inpatient participants.

The use of natural resources—mineral water, therapeutic peloids, and climate-based therapies—presents a safe, sustainable, and accessible complementary strategy for managing stress-related mental health conditions. These findings support the integration of balneotherapy into broader preventive mental health strategies, rehabilitation programs, and public wellness initiatives.

Policymakers and healthcare providers may consider incorporating balneotherapy into integrative care models, especially in settings with limited access to conventional psychological services. To support broader adoption, future research should focus on large-scale, long-term studies that compare BT with standard therapies, investigate cost-effectiveness, and explore its mechanisms of action. Development of clinical guidelines and protocol harmonization would further facilitate its implementation in mental health and wellness policy.

## Supplementary Information

Supplementary Material 1.

Supplementary Material 2.

## Data Availability

The datasets generated and analyzed during the current study are available from the corresponding author on reasonable request.
