# Supplementary material for: Effects of balneotherapy on stress, anxiety, and depression: results of a multicenter randomized controlled trial with six-month follow-up
Source: BMC Complement Med Ther. 2026 Mar 17;26:154. doi: 10.1186/s12906-026-05323-4 (PMC13107890; doi:10.1186/s12906-026-05323-4)
Supplement: Supplementary file 2 — Supplementary Material 2. [file 12906_2026_5323_MOESM2_ESM.docx]

The effect of changes of study outcomes compared with baseline

| 11ABT | | | | | 11ABTNT | | | | 11BTS | | | | 11C | | | | |
| --- | --- | --- | --- | --- | --- | --- | --- | --- | --- | --- | --- | --- | --- | --- | --- | --- | --- |
|  | Changes compared to the baseline | | | | Changes compared to the baseline | | | | Changes compared to the baseline | | | | Changes compared to the baseline | | | | |
|  | Mean (SD) | Mean difference | Effect size | p value | Mean (SD) | Mean difference | Effect size | p value | Mean (SD) | Mean difference | Effect size | p value | Mean (SD) | Mean difference | Effect size | p value | |
|  | Baseline | 95% CI |  |  | Baseline | 95% CI |  |  | Baseline | 95% CI |  |  | Baseline | 95% CI | |  |  |
|  | After |  |  |  | After |  |  |  | After |  |  |  | After |  |  |  |  |
| Saliva cortisol | 2.88 (1.54) | 0.72 | 0.367 | 0.009 | 3.75 (2.81) | 0.87 | 0.362 | 0.006 | 3.37 (2.40) | 0.64 | 0.206 | 0.117 | 2.54 (2.40) | -0.11 | | -0.046 | 0.748 |
|  | 2.16 (1.58) | 0.186 to 1.260 |  |  | 2.89 (1.87) | 0.254 to 1.477 |  |  | 2.73 (2.01) | -0.164 to 1.439 |  |  | 2.65 (1.89 | -0.766 to -0.553 | |  |  |
| Stress intensity | 6.14 (2.06) | 2.60 | 1.006 | <0.001 | 6.35 (2.66) | 2.89 | 0.783 | <0.001 | 6.00 (1.63) | 2.57 | 1.323 | <0.001 | 5.24 (2.07) | 1.18 | | 0.611 | <0.001 |
|  | 3.54 (2.05) | 1.952 to 3.255 |  |  | 3.46 (2.15) | 1,960 to 3.818 |  |  | 3.43 (1.43) | 2.076 to 3.072 |  |  | 4.06 (2.09) | 0.635 to 1.718 | |  |  |
| Stress management | 5.5 (1.67) | 1.11 | -0.409 | 0.002 | 6.41 (2.37) | -0,98 | -0.326 | 0.012 | 4.98 (1.63) | -1.89 | 0.863 | <0.001 | 6.71 (1.55) | 0.16 | | 0.092 | 0.516 |
|  | 6.61 (2.17) | -1.786 to -0.431 |  |  | 7.4 (2.45) | -1.744 to -0.224 |  |  | 6.87 (1.90) | -2.445 to -1.326 |  |  | 6.55 (1.69) | -0.325 to 0.639 | |  |  |
| PSS-10 | 16.44 (7.57) | 3.15 | 0.592 | <0.001 | 18.60 (7.52) | 2.76 | 0.324 | 0.002 | 18.70 (4.06) | 3.97 | 1.526 | <0.001 | 16.67 (5.18) | 1.35 | | 0.470 | 0.002 |
|  | 13.3 (5.67) | 1.786 to 4.509 |  |  | 15.84 (7.52) | 0.460 to 5.067 |  |  | 14.74(4.69) | 5.737 to 9.535 |  |  | 15.31 (5.54 | 0.544 to 2.162 | |  |  |
| FAS | 25.41 (8,01) | 4.13 | 0.566 | <0.001 | 28.40 (5.99) | 5.14 | 0.818 | <0.001 | 27.36 (5.64) | 4.75 | 0.794 | <0.001 | 23.59 (6.39) | -0.22 | | -0.04 | 0.775 |
|  | 21.28 (3.4) | 2.304 to 5.946 |  |  | 23.25 (5.54) | 3.560 to 6.726 |  |  | 22.61 (4.15) | 3.221 to 6.287 |  |  | 23.80 (4.95) | -1.724 to 1.293 | |  |  |
| STAIS | 9.21 (4.16) | 2.64 | 0.867 | <0.001 | 9.86 (4.53) | 2.31 | 0.703 | <0.001 | 8.35 (2.71) | 2.12 | 0.920 | <0.001 | 8.16 (2.24) | 0.45 | | 0.157 | 0.268 |
|  | 6.57 (2.26) | 1.870 to 3.400 |  |  | 7.66 (2.58) | 1.477 to 3.127 |  |  | 6.23 (1.93) | 1.522 to 2.711 |  |  | 7.71 (2.61) | -0.357 to 1.259 | |  |  |
| STAIT | 12.27 (4.14) | 3.47 | 1.431 | <0.001 | 13.03 (3.93) | 2.57 | 0.943 | <0.001 | 12.89 (3.21) | 3.85 | 1.493 | <0.001 | 9.94 (3.07) | 0.29 | | 0.134 | 0.342 |
|  | 8.80 (3.18) | 2.863 to 4.074 |  |  | 10.46 (3.57) | 1.884 to 3.258 |  |  | 9.03 (2.66) | 3.191 to 4.513 |  |  | 9.65 (2.86) | -0.323 to 0.911 | |  |  |
| CESD | 16.40 (14.44) | 12.49 | 0.866 | <0.001 | 22.51 (14.66) | 10.68 | 0.579 | <0.001 | 17.97 (9.70) | 10.93 | 1.332 | <0.001 | 11.76 (10.80) | 1.275 | | 0.162 | 0.253 |
|  | 3.90 (5.05) | 8.850 to 16.124 |  |  | 11.83 (13.44) | 6.034 to 15.331 |  |  | 7.03 (5.89) | 8.832 to 13.036 |  |  | 10.49 (8.80) | -0.939 to 3.488 | |  |  |
| WSAS | 13.13 (11.99) | 2.80 | 0.291 | 0.023 | 18.63 (9.83) | 8.43 | 0,734 | <0.001 | 15.89 (7.93) | 7.84 | 0.956 | <0.001 | 12.33 (10.43) | 0.45 | | 0.063 | 0.641 |
|  | 10.33 (12.27) | 0.400 to 5.194) |  |  | 10.21 (10.35) | 5.535 to 11.322 |  |  | 8.05 (7.21) | 5.737 to 9.935 |  |  | 11.88 (10.31) | -1.548 to 2.450 | |  |  |
| Working memory | -0.55 (1,23) | 0.087 |  | 0.557 | -0.27 (1.16) | 0.014 |  | 0.920 | -0.28 (1.35) | -0.388 |  | 0.117 | -0.84 (1.24) | -0.20 | |  | 0.258 |
|  | -0,63 (1.33) | -0.209 to 0.384 |  |  | -0.29 (1.04) | -0.265 to 0.2937 |  |  | -0.10 (1.31) | -0.879 to 0.103 |  |  | -0.58 (1.28) | -0.719 to 0.199 | |  |  |
| Working speed | -0.54 (1.37) | 0.032 |  | 0.834 | -0.71 (1.08) | -0.241 |  | 0.135 | -0.56 (1.23) | 0.364 |  | 0.157 | -0.39 (0.86) | 0.203 | |  | 0.253 |
|  | -0.57 (1.05) | -0.276 to 0.341 |  |  | -0.47 (0.94) | -0.559 to 0.078 |  |  | -0.47 (1.24) | -0.149 to 0.876 |  |  | -0.59 (1.30) | -0.152 to 0.557 | |  |  |
| Attention | -0.31 (0.92) | 0.023 |  | 0.919 | -0.28 (1.04) | -0.057 |  | 0.752 | -0.15 (0.80) | -0.029 |  | 0.925 | -0.11 (0.92) | -0.506 | | 0.067 | 0.705 |
|  | -0.34 (1.62) | -0.432 to 0.478 |  |  | -0.22 (0.78) | -0.418 to 0.303 |  |  | -0.16 (0.98) | -0.651 to 0.593 |  |  | -0.21 (1.27) | -0.201 to 1.274 | |  |  |
| Vision field | -0.40 (1.21) | -0.055 |  | 0.809 | -0.36 (0.92) | 0.146 |  | 0.533 | -0.41 (1.22) | 0.002 |  | 0.996 | -0.43 (1.21) | -0.433 | |  | 0.097 |
|  | -0.34 (1.47) | -0.507 to 0.397 |  |  | -0.51 (1.29) | -0.322 to 0.615 |  |  | -0.10 (1.17) | -0.639 to 0.642 |  |  | 0.01 (1.12) | -0.950 to 0.083 | |  |  |
| Usage of sedatives and hypnotics | 1.78 (1,10) | 0.22 | 0.2 | 0.109 | 1.53 (1,07) | 0.25 | 0.6 | <0.001 | 1.26 (0.72) | -0.07 | -0.1 | 0.419 | 1.63 (0.99) | 0.16 | | -0.1 | 0.337 |
|  | 1.57 (0.92) | -0.05 to 0.109 |  |  | 1.27 (0.83) | 0.136 to 0.373 |  |  | 1.32 (0.83) | -0.223 to 0.094 |  |  | 1.47 (1.04) | -0.175 to 0.502 | |  |  |
| The usage of antidepresants | 1.07^a^ | 0 | - | - | 1.42 | 0.06 | 0.2 | 0.083 | 1 | 0 | - | - | 1.08 | -0.08 | | -0.1 | 0.322 |
|  | 1.07^a^ |  |  |  | 1.36 | -0.007 to 0.117 |  |  | 1 |  |  |  | 1.16 | -0.246 to 0.083 | |  |  |
| Sleep quality | 5.80 (1.63) | -1.98 | -1.19 | <0.001 | 5.60 (2.87) | -2.25 | -0.802 | <0.001 | 4.61 (1.95) | -2.25 | -1.474 | <0.001 | 6.33 (1.67) | -0.14 | | -0.079 | 0.571 |
|  | 7.78 (1.80) | -2.417 to -1.549 |  |  | 7.86 (1.59) | -2.962 to -1.546 |  |  | 6,85 (1.74) | -2.636 to -1.856 |  |  | 6.47 (1.86) | -0.624 to 0.479 | |  |  |
| Integrative outcomes | 5.50 (1.63) | -1.91 | -0.806 | <0.001 | 4.98 (2.06) | -3.04 | -1.367 | <0.001 | 5.54 (1.95) | -1.35 | -0.778 | <0.001 | 6.74 (1.46 | 0.07 | | 0.05 | 0.724 |
|  | 7.41 (1.59) | -2.618 to -1.208 |  |  | 8.03 (1.47) | -3.603 to -2.482 |  |  | 6.90 (1.62) | -1.812 to -0.890 |  |  | 6.66 (1.56) | -0.335 to 0.479 | |  |  |

The correlation and t cannot be computed because the standard error of the difference is 0.

Abbreviations: 11ABT- 11 days (2-weeks) balneotherapy complex treatment group, 11ABTNT- 11 days (2-weeks) balneotherapy complex and nature therapy group, 11BTS- 11 days (2-weeks) balneotherapy complex inpatient group, 11C- 2-weeks control group; PSS-10- perceived stress scale; FAS- fatigue assessment scale; STAIS- The Spielberger State-*Trait Anxiety Inventory- state scale, STAIT-* The Spielberger State-*Trait Anxiety Inventory- trait scale; CESD-R-* Center for Epidemiologic Studies Depression *Scale* Revised; WSAS- work and social adjustment scale.
